# Exploring the therapeutic potential of “Xiaochaihu Decoction”: a systematic review and meta-analysis on the clinical effectiveness and safety in managing cancer-related fever

**DOI:** 10.3389/fphar.2024.1359866

**Published:** 2024-05-13

**Authors:** Zhijun Bu, Yaoyu Xu, Xian Zhou, Xuefeng Wang, Shuyuan Liu, Linyan Wang, Bei Yang, Xiaodie Zhou, Guanhang Lu, Jianping Liu, Zhaolan Liu

**Affiliations:** ^1^ Centre for Evidence-Based Chinese Medicine, Beijing University of Chinese Medicine, Beijing, China; ^2^ The First Clinical College, Hubei University of Traditional Chinese Medicine, Wuhan, China; ^3^ NICM Health Research Institute, Western Sydney University, Westmead, NSW, Australia; ^4^ School of Traditional Chinese Medicine, Hubei University of Chinese Medicine, Wuhan, China; ^5^ Acupuncture and Bone Injury College, Hubei University of Traditional Chinese Medicine, Wuhan, China

**Keywords:** Xiaochaihu Decoction, cancer-related fever, meta-analysis, systematic review, clinical effectiveness

## Abstract

**Objective:** This study aimed to conduct the first meta-analysis to comprehensively evaluate the clinical effectiveness and safety of Xiaochaihu Decoction in treating Cancer-related Fever (CRF).

**Methods:** Eight databases were systematically searched in September 2023. The risk of bias (ROB) 2.0 tool recommended by Cochrane Handbook was applied to evaluate the ROB of the included randomized controlled trials (RCTs). Additionally, the quality of evidence was assessed using the Grading of recommendations assessment, development and evaluation (GRADE) tool.

**Results:** We included 18 RCTs involving 1,424 patients. Compared to Western medicine or Xinhuang Tablets, Xiaochaihu Decoction significantly improved clinical effectiveness in CRF patients (risk ratio [RR] = 1.24, 95% confidence interval [CI]: 1.17, 1.32) and expedited the normalization of body temperature (mean difference [MD] = −5.29, 95%CI: −5.59, −4.99). It also demonstrated a reduction in tumor necrosis factor-α (TNF-α) levels (MD = −0.63, 95%CI: −0.84, −0.41) and an increase in IL-2 levels (MD = 1.42, 95%CI: −1.09, 1.74). Analysis of Karnofsky Performance Status (KPS) scores showed that the use of Xiaochaihu Decoction improved the quality of life in CRF patients (RR = 1.57, 95%CI: 1.11, 2.22) and reduced the incidence of adverse events. However, it is important to note that the majority of included studies showed “some concerns” in risk of bias based on ROB 2.0, and the evidence quality assessed by GRADE method was rated as “low”.

**Conclusion:** While this study suggests the clinical effectiveness and safety of Xiaochaihu Decoction in treating patients with CRF, confirming these findings will necessitate additional high-quality, large-scale RCTs in future research.

**Systematic Review Registration:** https://www.crd.york.ac.uk/prospero/, identifier CRD42023484068.

## Graphical Abstract

**Figure:**
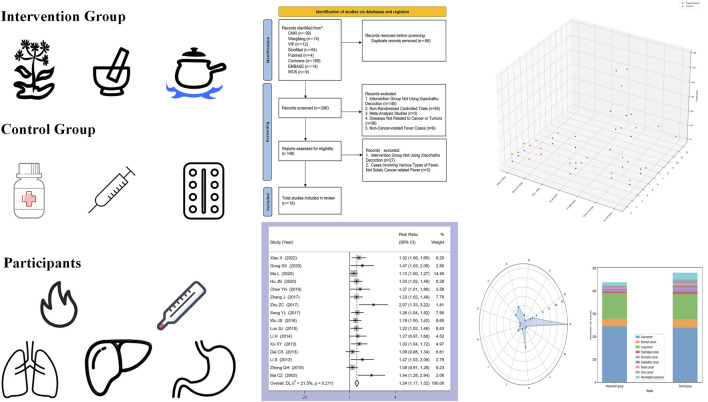
The icons in this image were sourced from Alibaba's icon library (https://www.iconfont.cn/).

## 1 Introduction

Cancer-related Fever (CRF) is defined as a fever directly associated with cancer, excluding instances caused by infection or treatment during tumor progression (H. Zhang et al., 2019). As a primary cause of non-infectious fever in patients with tumors, CRF is typically linked to the autonomous production of cytokines by cancer cells. These small proteins, endowed with diverse biological properties, can act as pyrogens, directly inducing fever by influencing the thermoregulatory centers in the hypothalamus (Toussaint et al., 2006).

Patients experiencing CRF generally display a persistent, low-grade fever, with temperatures ranging from 37.5°C to 38°C. The fever tends to manifest in an irregular or intermittent pattern (Cador-Rousseau, Cazalets-Lacoste and Grosbois, 2002). CRF is a tricky symptom, Warburg et al. (2008) proposed that tumor cells primarily ferment glucose, leading to an accelerated metabolism and an increase in body temperature (Majerović et al., 2008). Notably, for every 1°C increase in body temperature, there is a 13% rise in the basal metabolic rate, intensifying the consumption of nutrients such as sugar, protein, and fat in febrile patients, thereby augmenting stress on the body (S. Li, 2013). Furthermore, fever ca adversely impact digestion by impairing the secretion of digestive juices and reduces the activity of digestive enzymes, leading to symptoms such as appetite loss. It can also elevate heart and respiratory rates, imposing additional physical strain on cancer patients. Beyond the physical discomfort, CRF significantly affects patients’ psychological wellbeing, inducing anxiety, irritability, and other negative emotions, which severely impact their quality of life (Cai et al., 2021).

While non-steroidal anti-inflammatory drugs such as naproxen, indomethacin, and ibuprofen have demonstrated effectiveness in managing CRF (Johnson, 1996), the use of naproxen in CRF may lead to increased sweating, and some patients may experience a recurrence of the fever after discontinuing the drug (Chang and Gross, 1985). Prolonged use of naproxen for CRF can cause adverse reactions, including gastritis, gastrointestinal bleeding, and notably, thrombocytopenia. Furthermore, naproxen is contraindicated in CRF patients with cardiac, hepatic, and renal insufficiency (Süleyman, Demircan and Karagöz, 2007). Indomethacin provides an alternative for treating CRF, though its use warrants attention due to potential adverse effects on the gastrointestinal tract, central nervous system, and hematopoietic system (Hur et al., 2009).

Chinese botanical drugs have demonstrated unique potential in the treatment of tumors (Ling, Yue and Ling, 2014; Y; Wang et al., 2020; Y; Zhang, Lou, Wang, Yu and Shen, 2020), showcasing anti-tumor effects that can be beneficial in managing CRF (X. Z. Chen and Li, 2012). One such botanical drug formula is Xiaochaihu Decoction, initially described in traditional Chinese Medicine (TCM) classic *Treatise on Typhoid Fever* wrote by famous ancient TCM practitioner Zhang Zhongjing for febrile illnesses. This decoction comprises *Bupleurum chinense DC. [Apiaceae; Bupleuri radix]*, *Scutellaria baicalensis Georgi [Lamiaceae; Scutellariae radix]*, *Pinellia ternata (Thunb.) Makino [Araceae; Pinelliae rhizoma]*, *Panax ginseng C.A.Mey. [Araliaceae; Ginseng radix et rhizoma]*, *Ziziphus jujuba Mill. [Rhamnaceae; Jujubae fructus]*, *Glycyrrhiza uralensis Fisch. ex DC. [Fabaceae; Glycyrrhizae radix et rhizoma]*, and *Zingiber officinale Roscoe [Zingiberaceae; Zingiberis rhizoma recens]* (Sun, Gao and Qiao, 2023). The conventional method for preparing a decoction involves placing a specified quantity of medicinal metabolites into a pot, adding sufficient water to submerge them, and allowing them to macerate for 30–60 min. Following maceration, the mixture is heated to boiling point using a high flame before being lowered to a simmer for 20–45 min. The resultant decoction, yielding approximately 300–400 mL, should then be divided into two equal portions to be consumed in the morning and evening, respectively (J. Wang, 2020). Prolonged boiling or ‘decocting’ is the earliest and most popular method of preparing botanical drugs in the practice of traditional Chinese medicine (TCM) (H. Luo, Li, Flower, Lewith and Liu, 2012). Modern pharmacological studies have revealed that Xiaochaihu Decoction can mitigate inflammatory responses and alleviate liver fibrosis through multiple targets (S. J. Wang et al., 2023). Clinical trials further support its efficacy by showing that Xiaochaihu Decoction modulates the tumor microenvironment, inhibits tumor markers, prolongs survival, and holds a distinct advantage in treating CRF (Bao et al., 2020; W. Y; Zhang et al., 2022). Notably, Xiaochaihu Decoction exhibits a long-lasting effect, with a low rate of temperature rebound after discontinuation and few notable side effects. Its positive impact on relieving symptoms, controlling tumors, improving patients’ quality of life, and prolonging survival make it a promising therapeutic option (Z. J. Wu, 2004).

While randomized controlled trials (RCTs) in China have investigated the use of Xiaochaihu Decoction for CRF, a comprehensive systematic review of its clinical effectiveness and safety is currently lacking. Meta-analysis, recognized as the superior method for synthesizing research findings across various disciplines, becomes indispensable in overcoming the limitations of independent studies due to diverse conditions and potential biases in their results. These biases, when collectively analysed, may reveal a common underlying truth. Therefore, statistical analysis is crucial in exposing this truth. For the first time, our study systematically evaluated the clinical effectiveness and safety of Xiaochaihu Decoction in treating CRF using meta-analysis. Our objective is to provide evidence-based medical support for the safety and clinical effectiveness of Xiaochaihu Decoction in treating CRF. By employing this meta-analytical approach, we aimed to offer essential insights for clinicians (Puhan et al., 2014; Hernandez, Marti and Roman, 2020). The expected outcome will assist clinicians in devising appropriate treatment plans for CRF patients, ultimately benefiting these individuals.

## 2 Materials and methods

Our study was registered with International Prospective Register of Systematic Reviews (PROSPERO) under the registration number CRD42023484068. Following this, a meta-analysis was carried out in accordance with the guidelines stipulated in the Preferred Reporting Items for Systematic Reviews and Meta-Analyses (PRISMA) 2020 (Page et al., 2021), and the Cochrane Handbook for Systematic Reviews of Interventions (Higgins et al., 2011). The completed PRISMA checklist is provided in Supplementary Appendix SA.

### 2.1 Eligibility criteria

(1) Study Type: RCTs.
(2) Participants: All participants had a clear diagnosis of malignant tumors, irrespective of their age, gender, race, or disease duration.
(3) Intervention Measures: The intervention group implemented a regimen of Xiaochaihu Decoction alone or in combination with other Western medications. Supplementary Appendix SB describes the primary chemical metabolites of Xiaochaihu Decoction.
(4) Results: The primary outcomes include clinical effectiveness and time to body temperature normalization. Secondary outcomes encompass inflammatory cytokine levels, quality of life indices, and adverse events.
(5) Criteria for clinical effectiveness: A significant effect was observed when the body temperature returned to normal within 7 days of drug administration, with no recurrence observed within 3 days following the cessation of the drug. The drug was deemed effective if the body temperature dropped between 0.5°C and 1.5°C within 7 days of drug administration, even if it did not reach normal, and remained stable within 3 days of drug cessation. The drug was classified as ineffective if it did not meet the aforementioned criteria, or if the temperature decreased during drug administration but then recurred after the cessation of the drug. The clinical effectiveness rate was determined by the sum of the number of people who showed significant effects and those who were effectively treated, divided by the total number of people (Zhang et al., 2016).

### 2.2 Search strategy

A comprehensive search across multiple databases, including PubMed, Embase, Chinese SinoMed, Web of Science, Cochrane Library, China National Knowledge Infrastructure, Wanfang, and VIP. Both Chinese and English articles published from the inception of each database until 2023 were included. Our search strategy involved the utilization of Medical Subject Headings (MeSH) and free-text words. Detailed search strategies for each database can be found in Supplementary Appendix SC.

### 2.3 Research selection

The titles, abstracts, and full texts of each retrieved study underwent independently review for potential inclusion by four authors: YYX, SYL, BY, and XDZ. These authors were also responsible for extracting relevant data, including basic information and outcome data. Subsequently, ZJB and LYW independently verified the extracted information. If cases where inconsistencies were detected in the data extracted by the four authors, a group discussion was held among all authors to resolve these discrepancies. The criteria for data extraction included the first author, publication date, sample size (total number of participants in each intervention and control group, along with gender distribution), average age, tumor type, treatment in the intervention and control groups, treatment duration, clinical effectiveness, adverse events, and other outcome measures.

### 2.4 Risk of bias in individual studies

ZJB and LYW independently assessed the risk of bias (ROB), and any disagreements were resolved through discussion with ZLL. The ROB in each study was determined using the Cochrane Collaboration’s ROB Tool 2.0 (Higgins et al., 2011). Various factors contributing to research quality, such as the randomization process, deviations from intended interventions, missing outcome data, measurement of the outcome, and selection of the reported result, were meticulously examined. These factors were then categorized as having a low ROB, high risk of bias, or some concerns.

### 2.5 Statistical analysis

Stata version 17 and a random effects model for data merging were implemented. Continuous data were analysed using the Mean Difference (MD) and 95% Confidence Interval (CI), while categorical data were assessed using the Risk Ratio (RR) and 95% CI. Our analysis included subgroup analyses, sensitivity analyses, and the detection of publication bias using Egger’s test (Egger et al., 1997). For the incidence rate of adverse events, Python software and generated three-dimensional maps were employed to enhance data visualization.

### 2.6 Quality of evidence and consensus statement on the phytochemical characterisation of medicinal plant extracts (ConPhyMP) assessment

ZJB and YYX independently assessed the certainty of each outcome using the GRADEpro Guideline Development Tool (GRADEpro GDT), developed by the Grading of Recommendations, Assessment, Development, and Evaluation (GRADE) working group. In addition, SYL and LYW meticulously assessed the included studies, guided by the ConPhyMP checklist, to evaluate the phytochemical characterization of medicinal plant extracts (Heinrich et al., 2022). During the assessment process, any discrepancies were resolved through discussion or consulting the third reviewer ZLL.

## 3 Results

### 3.1 Database search

**FIGURE 1:**
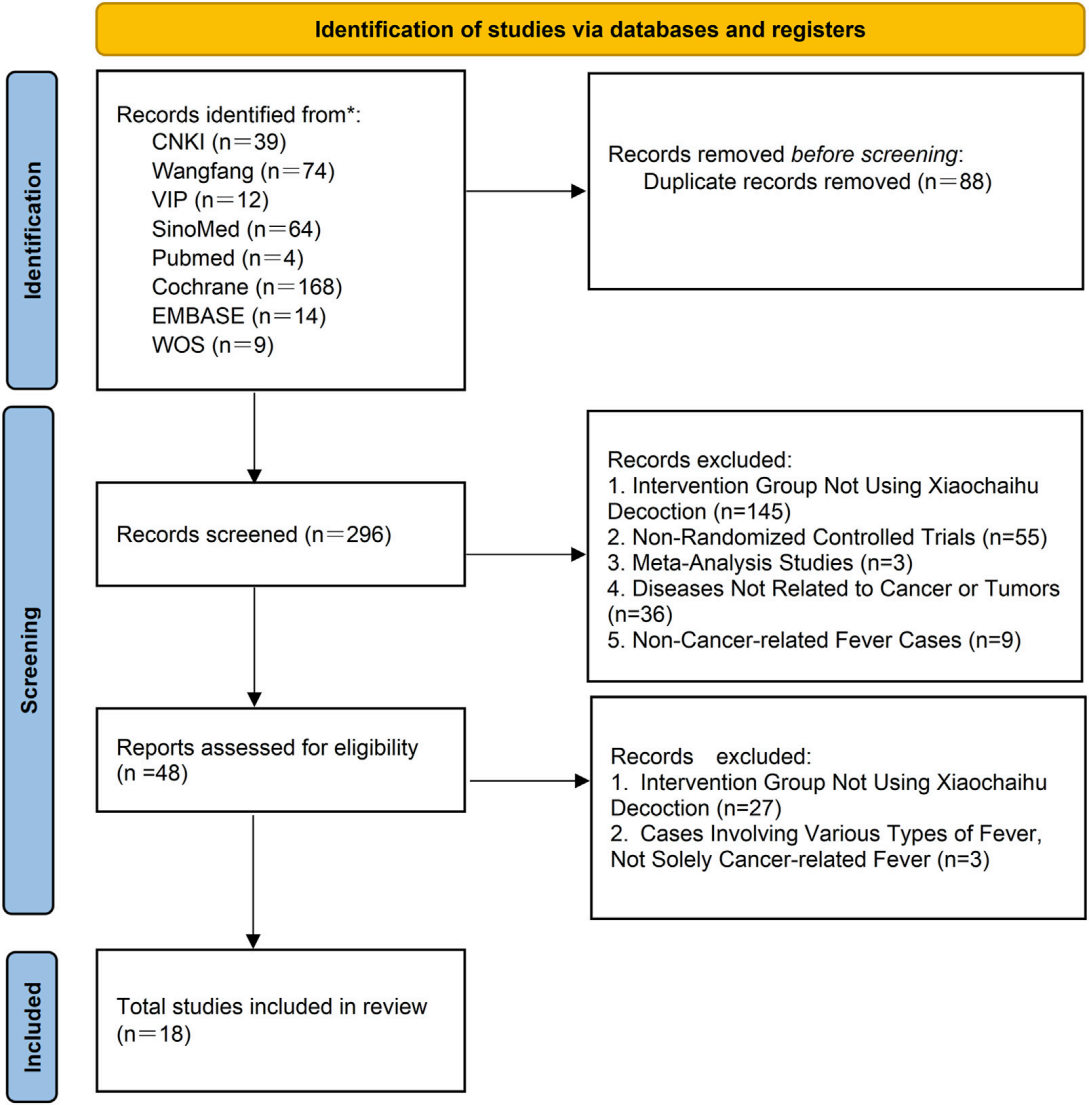
PRISMA flow chart of literature searching and screening. CNKI: China National Knowledge Infrastructure; SinoMed: the Chinese Biomedical Literature Database; WanFang: the WanFang Database; VIP: the Chinese Scientific Journals Full-Text Database; Embase Database: Excerpta Medica Database; WOS Database: Web of Science Database.

A total of 384 studies from eight databases were retrieved. After reexaminations, 88 studies were identified as duplicates using Endnote. Following preliminary screening of the remaining 296 studies, 248 were found not to meet the inclusion criteria. Subsequently, during a full-text screening of the remaining 48 studies, we identified 18 RCTs that met the criteria for inclusion in our article. A flowchart illustrating the study screening and selection process is present in Figure 1.

### 3.2 Study characteristics

**FIGURE 2:**
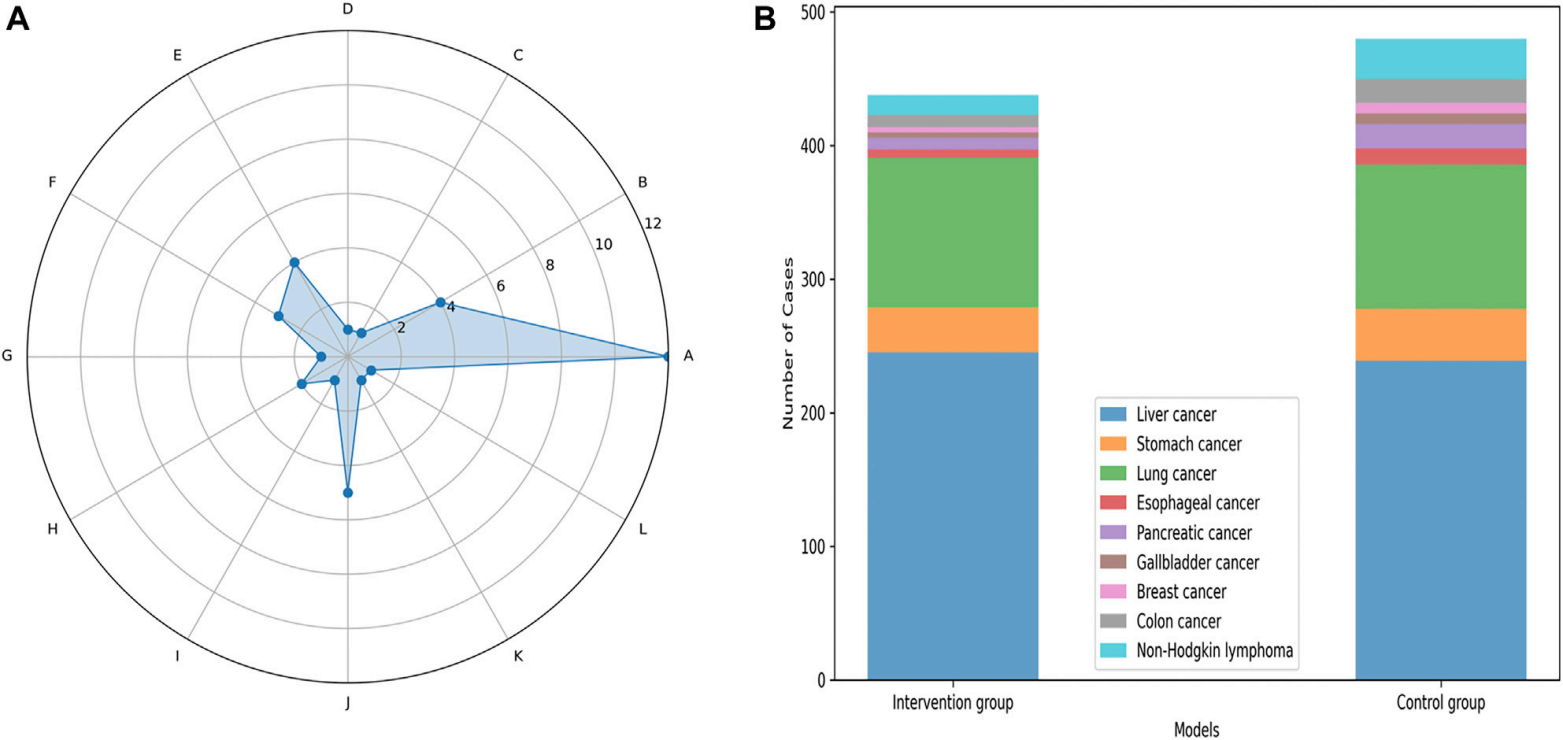
The basic information chart of the 18 studies included in the meta-analysis. The left image is a radar chart comparing the number of all treatment measures in the included literature. **(A)**, Xiaochaihu Decoction; **(B)**, Xiaochaihu Decoction plus Indomethacin; **(C)**, Xiaochaihu Decoction plus Xinhuang Tablets; **(D)**, Xiaochaihu Decoction plus radiotherapy and chemotherapy; **(E)**, Indomethacin; **(F)**, Naproxen Tablets; **(G)**, Xinhuang Tablets; **(H)**, Ibuprofen plus lysine-aspirin; **(I)**, Ibuprofen plus lysine-aspirin plus Indomethacin; **(J)**, Antipyretics plus antibiotic; **(K)**, Radiotherapy plus chemotherapy plus Indomethacin; **(L)**, Pure radiotherapy plus chemotherapy. The right image is a stacked bar chart comparing the number of tumor types involved in all intervention group and control group in the included studies.

In total, 18 studies published between 2002 and 2023 were included in our meta-analysis, comprising 1,424 patients with CRF. This included 711 patients in the control group, and 713 patients in the intervention group. Among the 18 included RCTs, liver and lung cancers were the most frequently observed types. The 18 RCTs encompassed 12 different treatment modalities for CRF in both traditional Chinese and Western medicine. Specifically, 12 studies utilized Xiaochaihu Decoction; five studies compared Xiaochaihu Decoction to Antipyretics plus antibiotics; three studies compared Xiaochaihu Decoction to Indomethacin; three studies compared Xiaochaihu Decoction with Naproxen Tablets; two studies compared Xiaochaihu Decoction to Ibuprofen plus lysine-aspirin; two studies compared Xiaochaihu Decoction to Xiaochaihu Decoction plus radiotherapy and chemotherapy; one study compared Xiaochaihu Decoction to Xiaochaihu Decoction plus Xinhuang tablets (Figure 2; Table 1). Furthermore, Supplementary Appendix SD includes the specific Gram weights of each botanical ingredient for a single dose of Xiaochaihu Decoction, as recorded in various studies.

**TABLE 1:** Basic characteristics of the included studies.

Study ID	Gender (male/Famale)	Sample size (I/C)	Age (mean Or range years)	Tumor type	Course (Mean or range, days)	Intervention *vs.* control	Daily dosage and frequency of medication	Outcomes
Xiao (2022)	43/37	I,40	I,56.24 ± 2.26	Liver cancer	I,7d	A VS H	I, 1 dose/d, 2 times/d	①②③④⑤
		C,40	C,56.39 ± 2.17	Stomach cancer	C,7d		C, NR	
				Lung cancer				
				Other cancer				
Gong et al. (2020)	28/22	I,25	I,54.4 ± 7.8	NR	I,7d	A VS E	I, 1 dose/d, 2 times/d	①②⑤
		C,25	C,55.1 ± 8.6		C, NR		C, 100 mg/d, 1 time/d	
Ma et al. (2020)	76/52	I,64	I,60.65 ± 5.82	NR	I,7d	A VS F	I, 1 dose/d, 2 times/d	①③⑤
		C,64	C,60.08 ± 5.89		C,7d		C, 0.25g/time, 2∼3times/d	
Hu et al. (2020)	59/47	I,53	I,60.87 ± 9.14	Liver cancer	I,7d	A VS I	I, 1 dose/d,2 times/d	①②③④⑤
		C,53	C,61.05 ± 8.78	Stomach cancer	C,7d		C, NR	
				Lung cancer				
				Non-Hodgkin lymphoma				
				Other cancer				
Lin and Zhao (2020)	42/34	I,38	I,58.82 ± 4.16	Lung cancer	I,42d	B vs. L	I, 1dose/d, 2times/d	④⑤
		C,38	C,57.74 ± 3.87		C,42d		C, Multiwis paclitaxel 20mg/branch	
							cisplatin 30mg/bottle venous drip	
Chen (2019)	41/19	I,30	I,66.35 ± 5.34	NR	I,7d	B VS E	I, 1 dose/d, 2 times/d	①②⑤
		C,30	C,66.31 ± 5.40		C, NR		C, 100mg, 1 time/d	
Zhang et al. (2017)	79/69	I,75	I,65.3 ± 5.5	NR	I,7d	A VS F	I, 1 dose/d,2 times/d	①④
		C,73	C,62.6 ± 5.9		C,7d		C, 0.25g/time, 2–3 times/d	
Zhu and Zong (2017)	64/20	I,42	I,58.5	Liver cancer	I,14d	C VS G	I, 1 dose/d, 2 times/d	①
		C,42	C,57		C,14d		C, 2tablets/time	
							3 times/d	
Song and Qi (2017)	33/37	I,35	I,50.0 ± 19.0	NR	I,10d	A VS J	I, 1 dose/d, 2 times/d	①
		C,35	C,50.0 ± 19.0		C, NR		C, NR	
Wu (2016)	36/28	I,32	I,53.4 ± 5.0	Liver cancer	I,14d	B VS E	I,1dose/d, 2times/d	①
		C,32	C,54.2 ± 5.5		C, NR		C,100mg/time	
							1time/d	
Luo (2015)	37/31	I,34	I,62.87 ± 2.23	Liver cancer	I,7d	A VS J	I, 1 dose/d, 2 times/d	①②⑤
		C,34	C,62.51 ± 2.65	Stomach cancer	C, NR		C, NR	
				Lung cancer				
				Esophageal cancer				
				Pancreatic cancer				
Li et al. (2014)	38/26	I,32	I,62.5 ± 7.4	Liver cancer	I,7d	A VS J	I, 1 dose/d, 2 times/d	①②
		C,32	C,62.5 ± 7.4	Stomach cancer	C,7d		C, NR	
				Lung cancer				
				Esophageal cancer				
				Pancreatic cancer				
Xu (2013)	35/25	I,30	I,61.87 ± 7.05	Liver cancer	I,10d	A VS J	I, 1 dose/d, 2 times/d	①②
		C,30	C,61.27 ± 7.44	Stomach cancer	C,10d		C, NR	
				Lung cancer				
				Esophageal cancer				
				Pancreatic cancer				
Dai et al. (2013)	58/38	I,48	I,70.2 ± 8.6	Liver cancer	I,7d	D VS K	I, 1 dose/d, 2 times/d	①
		C,48	C,72.6 ± 4.7	Stomach cancer	C,7d		C, 0.5 grain/d	
				Lung cancer				
				Pancreatic cancer				
				Gallbladder cancer				
				Colon cancer				
Li et al. (2013)	51/9	I,30	I,57.07 ± 11.08	Liver cancer	I,7–14d	B VS E	I, 1 dose/d,2 times/d	①
		C,30	C,58.97 ± 9.76		C,7–14d		C, 100mg/time	
Zheng et al. (2010)	39/21	I,30	I,63.2 ± 5.6	NR	I,10d	A VS J	I, 1 dose/d,2 times/d	①
		C,30	C,59.6 ± 8.2		C,10d		C, NR	
Ma et al. (2002)	72/18	I,45	I,51.5	Liver cancer	I, NR	A VS F	I, 1 dose/d,2 times/d	①
		C,45	C,53.3		C, NR		C, 50mg–100mg/time,2times/d	
Peng (2018)	33/27	I,30	I,53.47 ± 10.15	Liver cancer	I,7d	A VS H	I, 1 dose/d, 2 times/d	①④
		C,30	C,55.60 ± 8.83	Lung cancer	C,7d		C, 0.3g/time	
				Breast cancer			Up to twice a day	
				Colon cancer				
				Non-Hodgkin lymphoma				

Annotation for Table 1: “I" refers to the intervention group, and “C" refers to the control group. In the ‘Intervention vs. control’ column, the specific meanings of all treatment measures are as follows: A, xiaochaihu decoction; B, xiaochaihu decoction plus indomethacin; C, xiaochaihu decoction plus xinhuang tablets; D, xiaochaihu decoction plus radiotherapy and chemotherapy; E, indomethacin; F, naproxen tablets; G, xinhuang tablets; H, Ibuprofen plus lysine-aspirin; I, Ibuprofen plus lysine-aspirin plus Indomethacin; J, antipyretics plus antibiotic; K, radiotherapy plus chemotherapy plus indomethacin; L, Pure radiotherapy plus chemotherapy. In the ‘Outcomes’ column, the meanings are as follows: ① represents Clinical effectiveness, ② represents Time to normalize body temperature, ③ represents Inflammatory cytokines levels, ④ represents Quality of life indices, ⑤ represents Adverse events.

### 3.3 Meta-analysis

#### 3.3.1 Clinical effectiveness

**FIGURE 3:**
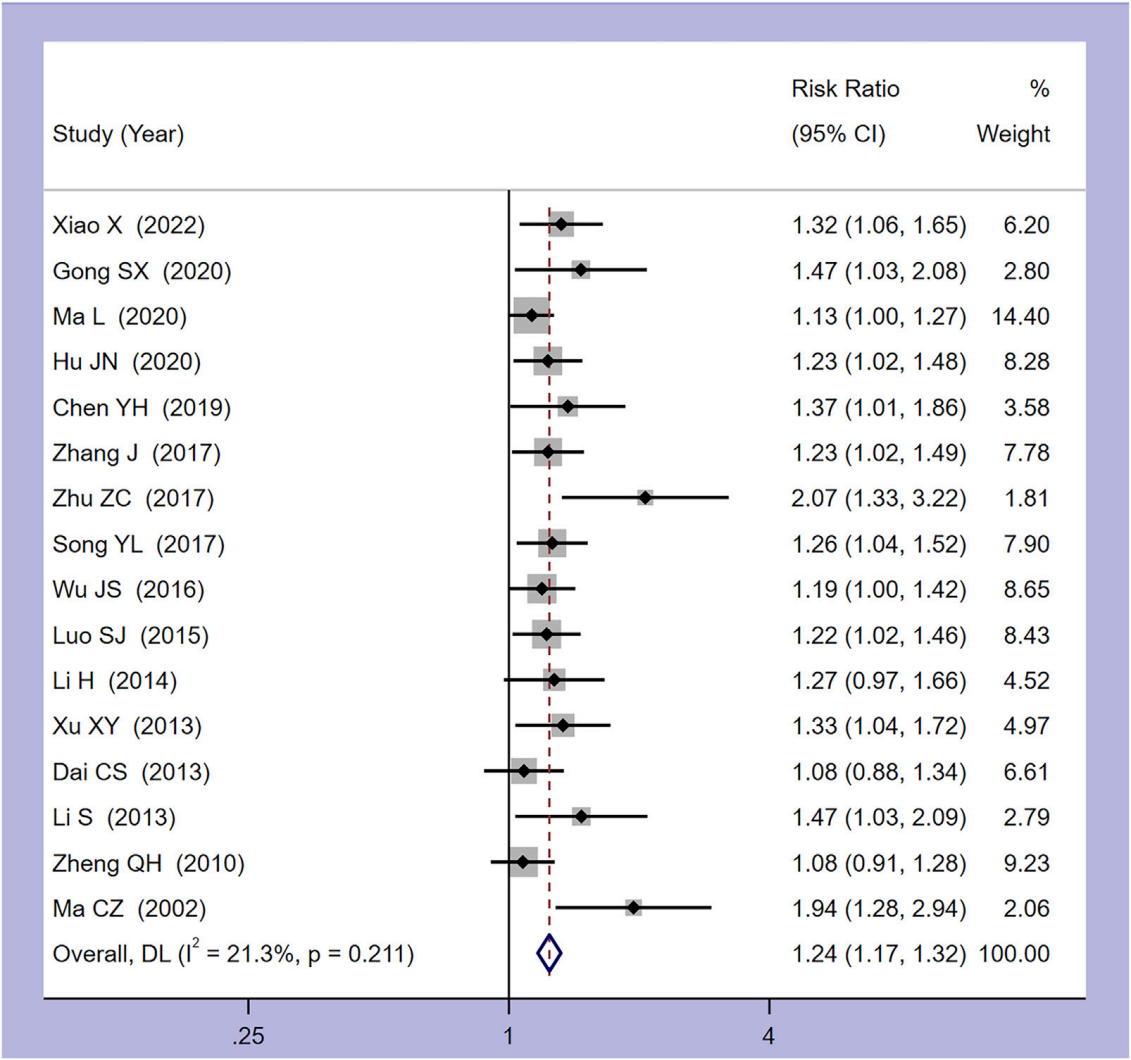
Forest plot of the meta-analysis on clinical effectiveness. RR: risk ratio, MD: mean difference, CI: confidence interval.

Among the 18 studies included, 17 utilized clinical effectiveness as an outcome measure. One study employed TCM symptom score as a clinical effectiveness criterion, while the remaining studies considered the return of body temperature to normal as an effective treatment criterion (Lin and Zhao, 2020). Consequently, a meta-analysis was performed on the 16 studies with a uniform clinical effectiveness criterion, utilizing a random-effects model. The analysis revealed a heterogeneity (I^2^ = 21.3%, *p* = 0.211). Figure 3 indicates that Xiaochaihu Decoction significantly enhances the clinical effectiveness in patients with CRF compared to the control group, with statistical significance (RR = 1.24, 95% CI: 1.17, 1.32).

#### 3.3.2 Time to normalize body temperature

**FIGURE 4:**
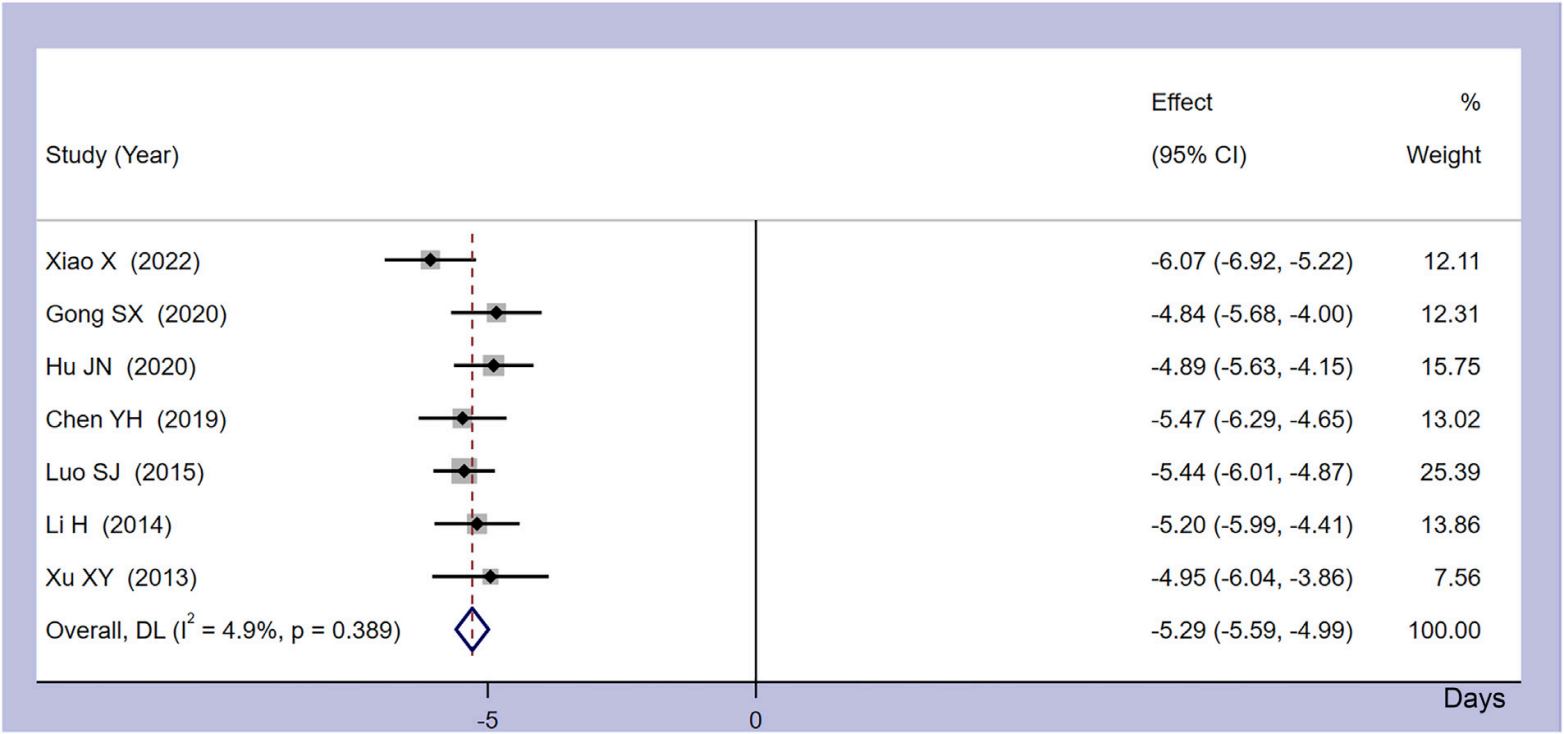
Forest plot of the meta-analysis on time to normalize body temperature. RR: risk ratio, MD: mean difference, CI: confidence interval.

Seven studies provided details on the time taken for the body temperature of CRF patients to normalize. As shown in Figure 4, the meta-analysis combined model demonstrated minimal heterogeneity, with I^2^ = 4.9% and *p* = 0.389. The collated results indicated that, compared to the control group, Xiaochaihu Decoction could expedite the normalization of body temperature in cancer patients, with a mean difference (MD) of −5.29 (95% CI: −5.59, −4.99). Therefore, the use of Xiaochaihu Decoction in CRF patients significantly reduced the average time to normalization of body temperature by 5.29 days.

#### 3.3.3 Inflammatory cytokines levels

Three studies investigated the serum levels of inflammatory markers, namely, tumor necrosis factor-α (TNF-α), interleukin-1β (IL-1β), interleukin-2 (IL-2) and cyclooxygenase-2 (COX-2), in patients with CRF. Since more than two studies provided data on TNF-α and IL-2 separately, a meta-analysis was conducted on these two markers. Our findings (Supplementary Appendix SE1) indicated that Xiaochaihu Decoction reduced the TNF-α levels to 0.63 g/L (MD = −0.63, 95%CI: −0.84, −0.41), and increased IL-2 levels to 1.42 g/L (MD = 1.42, 95%CI: −1.09, 1.74) compared to the control group. Only one investigation, Ma et al., 2020, reported pre- and post-treatment changes in IL-1β and COX-2 levels in CRF patients (Ma et al., 2020). According to the data comparing patients before and after treatment, Xiaochaihu Decoction resulted in decreased IL-1β and COX-2 levels in patients with CRF.

#### 3.3.4 Quality of life indices

Three studies assessed quality of life indices in patients with CRF, utilizing the Karnofsky Performance Status (KPS) score, while another study used the Quality of Life (QOL) score. The analysis of KPS scores (Supplementary Appendix SE2) suggested that the administration of Xiaochaihu Decoction could significantly enhance the quality of life in patients with CRF (RR = 1.57, 95%CI: 1.11, 2.22). In a separate study by Xiao X (2022), QOL scores were documented (Xiao, 2022). The results indicated that the scores for somatic, psychological, physiological, and social functioning were higher in the group treated with Xiaochaihu Decoction as compared to the control group.

#### 3.3.5 Adverse events

**FIGURE 5:**
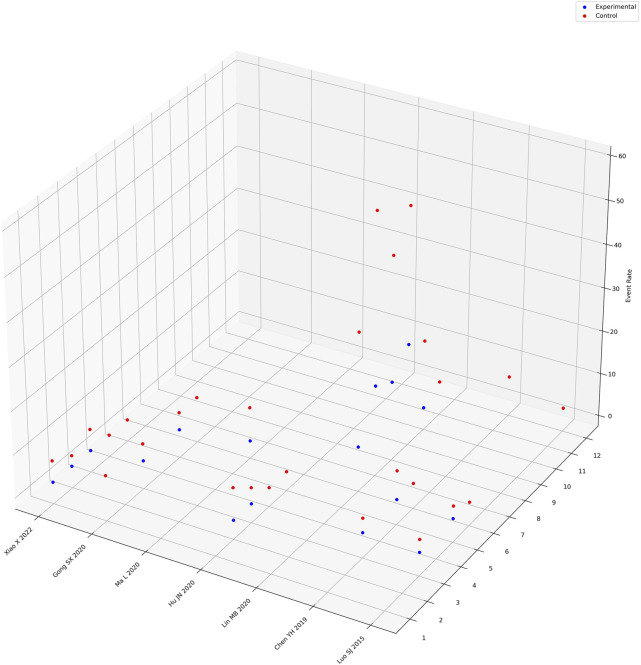
Three-dimensional guidance diagram of adverse event incidence rates. The X-axis represents the names of randomized controlled trials recording adverse reactions, the Y-axis represents the names of adverse reaction events, where “1” represents nausea, “2” represents diarrhea, “3” represents dizziness/headache, “4” represents gastrointestinal bleeding, “5” represents palpitations, “6” represents abdominal bloating/pain, “7” represents leukopenia, “8” represents thrombocytopenia, “9” represents decreased hemoglobin, “10” represents abnormal liver function, “11” represents abnormal kidney function, “12” represents decreased appetite. The Z-axis represents the proportion of adverse reaction events.

Seven studies explicitly reported adverse events in both the intervention and control groups, encompassing various issues such as nausea, diarrhea, dizziness/headache, gastrointestinal bleeding, palpitations, abdominal bloating/pain, leukopenia, thrombocytopenia, decreased hemoglobin, abnormal liver function, abnormal kidney function, and decreased appetite. A meta-analysis was conducted on the occurrence of abdominal bloating/pain (from four studies) (Hu, Ma, Xu and Wang, 2020; Lin and Zhao, 2020; L; Ma et al., 2020; Xiao, 2022), palpitations (from five studies) (Y. H. Chen, 2019; Gong, Xue and Qeng, 2020; Hu et al., 2020; S. J; Luo, 2015; Xiao, 2022), and dizziness/headache (from five studies) (Y. H. Chen, 2019; Gong et al., 2020; Hu et al., 2020; S. J; Luo, 2015; Xiao, 2022). The data analysis (Supplementary Appendix SE3) revealed that the administration of Xiaochaihu Decoction decreased the incidence of abdominal bloating/pain (RR = 0.23, 95%CI: 0.09, 0.58), palpitations (RR = 0.40, 95%CI: 0.13, 1.26), and dizziness/headache (RR = 0.42, 95%CI: 0.15, 1.17) compared to the control group. Furthermore, a three-dimensional guide diagram (Figure 5) was used to illustrate the incidence of adverse events across the seven studies. The diagram suggested that the incidence of adverse events was higher in the control group than in the intervention group treated with Xiaochaihu Decoction.

### 3.4 Subgroup analysis

Subgroup analyses were conducted on the endpoints of clinical effectiveness and time to normalize body temperature, stratified by different treatment types. Regarding clinical effectiveness, statistically significant differences were observed in all intervention groups, except for comparisons Xiaochaihu Decoction *versus* Naproxen Tablets (Supplementary Appendix SE4). Concerning the endpoint of time to normalize body temperature, all intervention groups exhibited a statistically significant difference compared to their respective control groups (Supplementary Appendix SE4).

### 3.5 Sensitivity analysis and publication bias assessment

**FIGURE 6:**
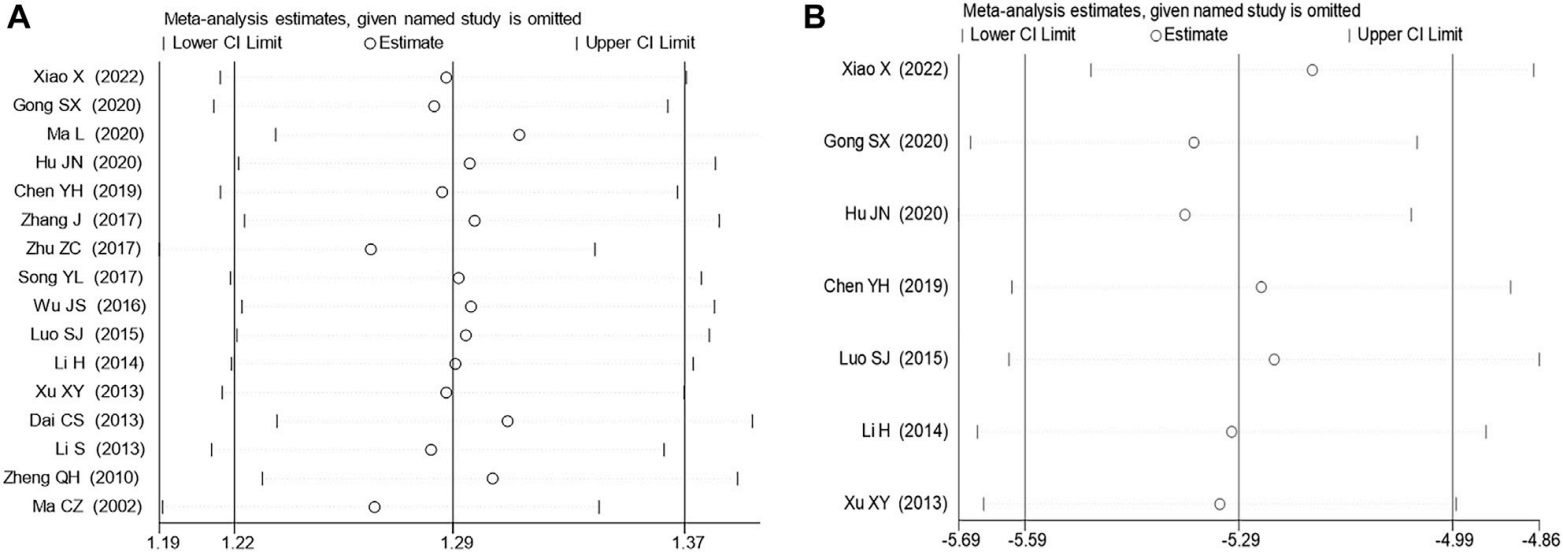
Sensitivity analysis on clinical effectiveness and time to normalize body temperature. **(A)** Clinical effectiveness, **(B)** Time to normalize body temperature.

**FIGURE 7:**
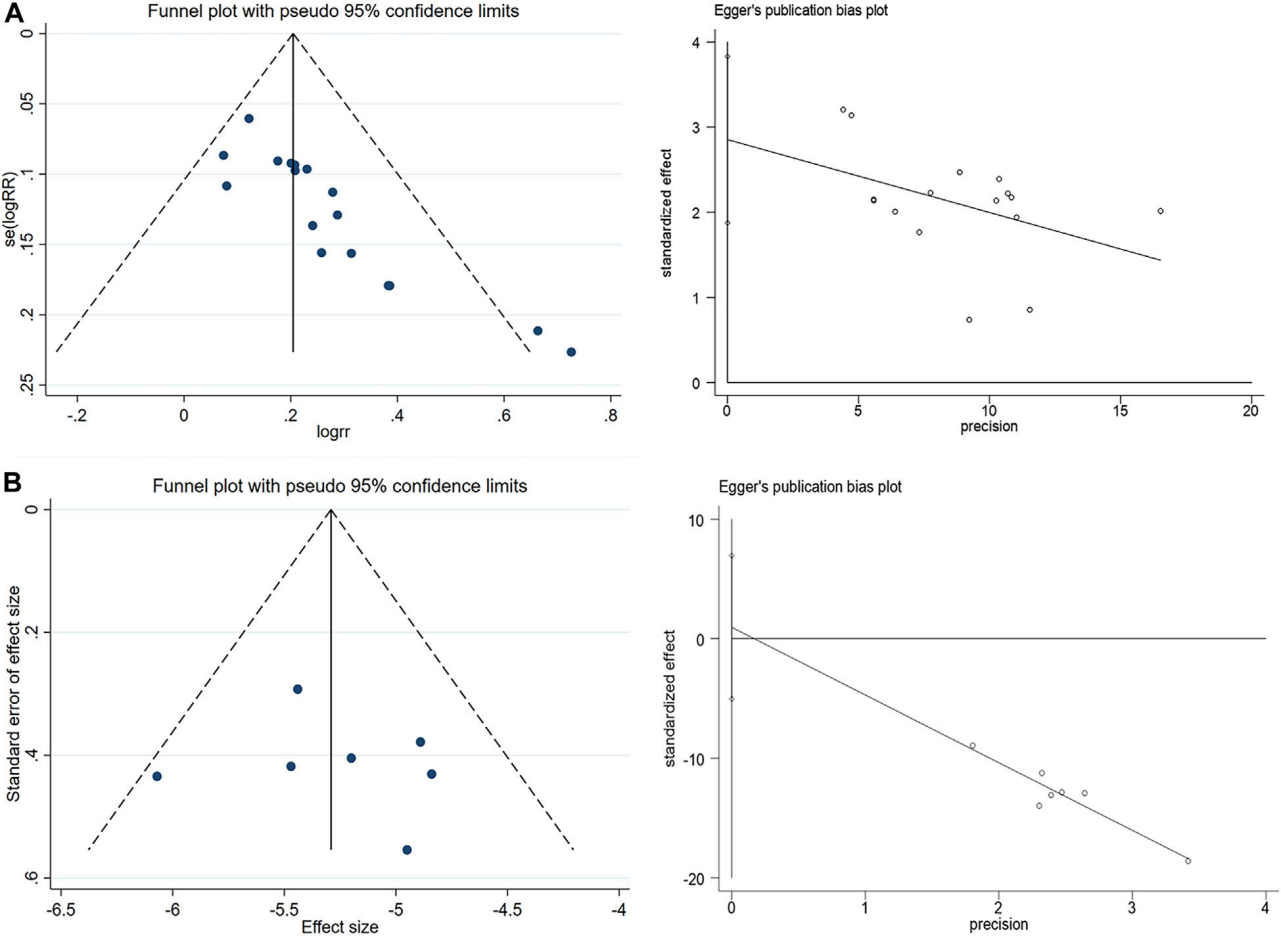
Publication bias detection and Egger’s test for clinical effectiveness and time to normalize body temperature. **(A)** Clinical effectiveness, **(B)** Time to normalize body temperature.

A random-effects model was employed for the meta-analysis, revealing relatively low heterogeneity in clinical effectiveness (I^2^ = 21.3%, *p* = 0.211). However, sensitivity analyses (Figure 6) and the detection of publication bias (Figure 7) identified two studies - Zhu and Zong, (2017) (Zhu and Zong, 2017) and Ma and Cheng et al. (2002) (Ma and Cheng, 2002) - that contributed to greater heterogeneity. Applying Egger’s test (Figure 6) yielded a significant result (*p* = 0.000), leading to the exclusion of these two studies. Subsequent meta-analysis demonstrated considerably reduced heterogeneity in the clinical effectiveness (I^2^ = 0.0%, *p* = 0.797). It was observed that the Xiaochaihu Decoction significantly increased clinical effectiveness in patients with CRF compared to the control group (RR = 1.21, 95%CI: 1.14, 1.27) (Supplementary Appendix SE5). Regarding the time-to-normalize-body-temperature outcome, sensitivity analysis demonstrated robust results, with a symmetrical funnel plot and a non-significant Egger’s test (*p* = 0.7). This provided further evidence of the robustness of the time-to-normalize-body-temperature outcome.

### 3.6 ROB analysis

**FIGURE 8:**
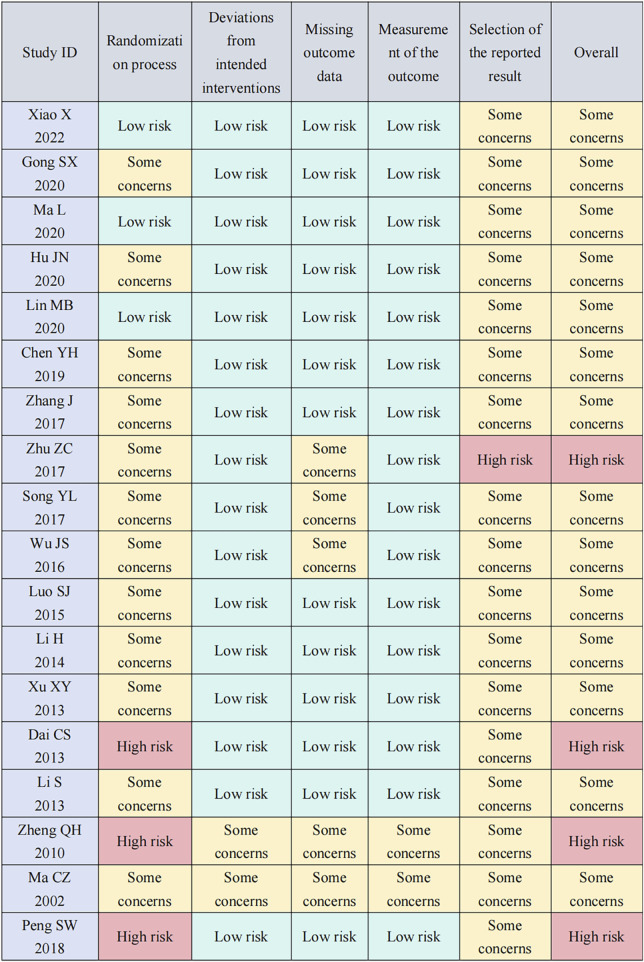
Risk of bias summary of the 18 included randomized controlled trials.

We assessed eighteen studies for ROB, and result showed that four were assessed as high-risk and fourteen as having some concerns. In the randomization process, three studies that employed a randomized table of numbers method for patient assignment were rated as low-risk (Lin and Zhao, 2020; L; Ma et al., 2020; Xiao, 2022), while the other three studies that assigned patients based on the order of arrival were rated as high-risk (Zheng, Dou and Wang, 2010; Dai, Wang, Chen and Shen, 2013; Peng, 2018). The remaining studies in this field were evaluated as having some concerns. In the field addressing deviations from intended interventions and measurement of outcomes, none of the studies explicitly mentioned blinding. Concerning statistical analysis, sixteen studies detailed their methods and were thus rated as low-risk. The remaining two studies, which did not specify their statistical analysis methods, were evaluated as having some concerns (C. Z. Ma and Cheng, 2002; Zheng et al., 2010). In the field addressing missing outcome data and partial outcome reporting, five studies reported only one outcome measure, leading to a rating of some concerns (C. Z. Ma and Cheng, 2002; Song and Qi, 2017; J. S; Wu, 2016; Zheng et al., 2010; Zhu and Zong, 2017). Conversely, the remaining studies presented comprehensive data with multiple outcome indicators, earning them a low-risk rating. In the field of the reported result, one study mentioned multiple outcome measures in the methods section but reported only one outcome, resulting in a high-risk rating (Zhu and Zong, 2017). Furthermore, the studies were unregistered, making it impossible to assess the consistency between their actual results and any previously planning. Consequently, they were rated as having some concerns (Figure 8).

### 3.7 Grade evidence evaluation and ConPhyMP assessment

**FIGURE 9:**
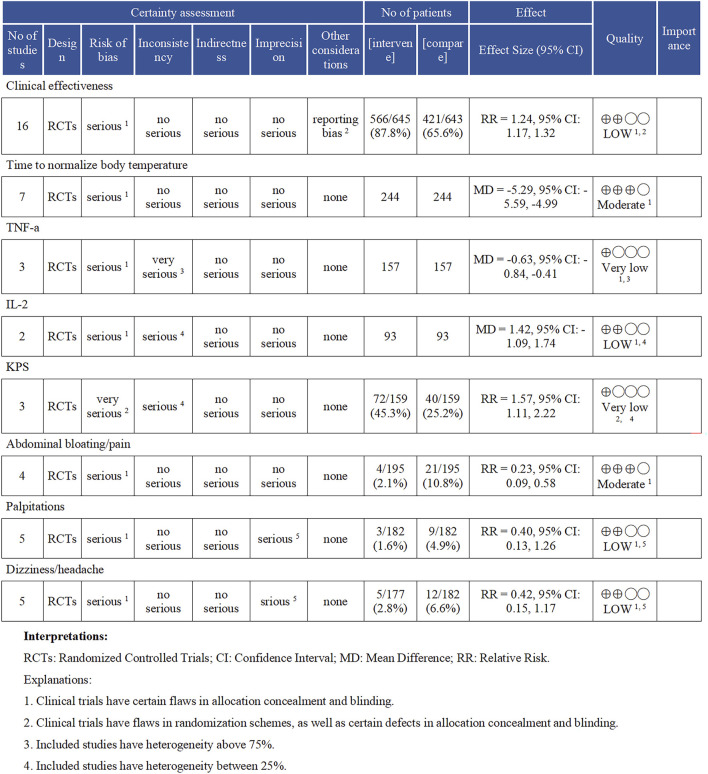
Results of the GRADE evidence evaluation. TNF-α (Tumor Necrosis Factor-alpha), IL-2 (Interleukin-2), and KPS (Karnofsky Performance Status).

Each meta-analysis result underwent meticulous evaluation for GRADE evidence, as detailed in Figure 9. The outcome measures of Time to Normalize Body Temperature and Abdominal Bloating/Pain were assessed as having moderate evidence, according to the GRADE evaluation criteria. The outcomes for clinical effectiveness, IL-2, palpitations, and dizziness/headache were classified as having low evidence. Meanwhile, the TNF-α and KPS scores were evaluated as having very low evidence. We assessed the 18 RCTs using the ConPhYMO tool statement report. We found that only item one applied to confirm that the species or botanical drug under investigation was covered in a monograph in one of the national or regional pharmacopoeias (Supplementary Appendix SF). We speculated that this might be related to the TCM decoction method.

## 4 Discussion

In China, Xiaochaihu Decoction is widely used and has shown effectiveness in treating acute upper respiratory tract infections (AURTI), as substantiated by a meta-analysis conducted by Yan, LJ et al. (2021) (Yan et al., 2021). Additionally, Wang, SJ et al. (2023), employing network pharmacology and *in vitro* experiments, have proposed Xiaochaihu Decoction as a potential therapeutic agent for liver fibrosis (S. J. Wang et al., 2023). Recent studies have investigated the efficacy of Xiaochaihu Decoction in managing CRF through RCTs. Preliminary findings suggested that patients with CRF might benefit from Xiaochaihu Decoction. However, as of now, no comprehensive reviews or meta-analyses have been conducted to evaluate the effectiveness and safety of Xiaochaihu Decoction in this context of CRF.

Our study aimed to further substantiate the potential and safety of Xiaochaihu Decoction in managing CRF. Through a systematic review of eight domestic and international databases, 18 RCTs were included, involving 1,424 patients with CRF. The most prevalent cancers among these patients were liver and lung cancer, suggesting that Xiaochaihu Decoction may be particularly effective for patients with these types of cancer. The 18 RCTs encompassed 12 different treatment modalities for CRF in both traditional Chinese and Western medicine. Frequently used treatments included Xiaochaihu Decoction, antipyretics in conjunction with antibiotics, indomethacin, and ponatinib. Our meta-analysis corroborated that Xiaochaihu Decoction significantly ameliorated the clinical effectiveness rate in patients with CRF (RR = 1.24, 95%CI: 1.17,1.32). Moreover, Xiaochaihu Decoction expedited the normalization of body temperature by an average of 5.29 days compared to the control group. Additionally, it significantly influenced TNF-α, IL-1β, and COX-2 levels, providing valuable insights to its therapeutic impact.

TNF-α, predominantly produced by mononuclear phagocytes, has the capability to induce the secretion of substantial amounts of cytokines, including IL-1 and IL-1β. These cytokines, acting as endogenous pyrogens, influence the body’s temperature center, resulting in fever (Yang, Song, Hu, Hua and Li, 2009). IL-2 plays a crucial role in modulating the body’s immune response, promoting the activation and proliferation of T cells, NK cells, and B cells. It enhances cytokine and corresponding antibody levels while exerting inhibitory effects on tumor cells (B. H. Zhang et al., 2011). COX-2, an inducible enzyme, can participate in tumor development through various pathways. Its overexpression can lead to increased PGE2, causing fever and inducing high expression of leukocyte interleukins (Shen and Feng, 1989). Our data analysis revealed that Xiaochaihu Decoction reduced the levels of inflammatory factors TNF-α, IL-1β and COX-2, while increasing IL-2 levels in patients with CRF. Quality of life indices (KPS and QOL) suggested that Xiaochaihu Decoction significantly improved the quality of life indices for patients with CRF. Furthermore, the meta-analysis and three-dimensional diagram analysis results showed a lower incidence of adverse events in the Xiaochaihu Decoction group compared to the control group, implying that Xiaochaihu Decoction is both effective and safe in treating CRF.

Xiao Chaihu Decoction, a TCM formula, is frequently employed to manage febrile conditions. Its primary composition is *Bupleurum chinense DC. [Apiaceae; Bupleuri radix]* contains complex chemical metabolites, including saponins, volatile oils, flavonoids, and polysaccharides. These metabolites play a pivotal role in its pharmacological effects, contributing to immune regulation, anti-depression, anti-tumor, and anti-inflammatory properties (Kato et al., 1994; Zell and Chang, 2005; Foggo and Cavenagh, 2015; Fang, Wang, Yang, You and Xing, 2022; Sun et al., 2023). *Bupleurum chinense DC. [Apiaceae; Bupleuri radix]* and *Scutellaria baicalensis Georgi [Lamiaceae; Scutellariae radix]* constitute the key medicinal pair in Xiao Chaihu Decoction. Their primary active metabolites, quercetin, bergenin, isorhamnetin, baicalein, and wogonin, demonstrate the ability to inhibit the expression of PTGS2 and PTGS1 genes, consequently reducing prostaglandin synthesis. Moreover, they suppress the expression of PRSS1, Caspase-3, and AKT1 genes, contributing to tumor growth inhibition. These metabolites function by blocking the activation of cancer-related pathways, such as the TNF and IL-17 pathways, ultimately reducing the release of fever-inducing cytokines like IL-1, IL-6, and TNF-α.

Quercetin, a flavonoid metabolite, is believed to exhibit a spectrum of beneficial effects, including antioxidant, antiviral, anti-inflammatory, anti-tumor, and immune regulatory effects (Liu and Liu, 2020). Bergenin considered a safe and potential radiosensitizer, enhances radiation damage to tumor cells both *in vitro* and *in vivo* by inhibiting the AKT/PI3K and ERK pathways and activating the mitochondrial apoptosis pathway (Pei et al., 2022). Inducible PTGS2 contributes to the production of inflammatory prostaglandins, and its upregulation is linked with increased cell adhesion, phenotype alterations, anti-apoptosis, and tumor angiogenesis (X. H. Wang et al., 2021). The serine/threonine kinase encoded by AKT1 prevents cell apoptosis by phosphorylating and inactivating metabolites of the apoptosis mechanism (G. L. Li, Liu, Xie, Yu and Hu, 2020). Additionally, the protein encoded by IL-6 is proven to be an endogenous pyrogen capable of inducing fever in patients with autoimmune diseases or infections (Kang et al., 2023).

Jiang and Wang, (2015) conducted an observational study on the clinical effectiveness of Xiao Chaihu Decoction in 47 patients with CRF. The study revealed satisfactory results in managing CRF, with no severe adverse reactions observed during the treatment, suggesting its potential for broader use (Jiang and Wang, 2015). Similarly, Zhang, (2013) employed Xiao Chaihu Decoction in patients with CRF and found that 83.3% of patients reported symptom relief and maintained a body temperature below 37.0°C for three consecutive days (L. K. Zhang, 2013). However, its important to note that current empirical studies on Xiao Chaihu Decoction for treating CRF primarily rely on small sample sizes. Therefore, larger-scale and more rigorously designed clinical trials are essential to validate the efficacy and safety of Xiao Chaihu Decoction. Encouraging more researchers and institutions to participate in empirical investigation of Xiao Chaihu Decoction for treating CRF will contribute valuable evidence to support its application in this context.

Our research possesses several noteworthy strengths. Notably, we conducted a pioneering comprehensive meta-analysis of previously published RCTs. The outcomes, characterized by low heterogeneity, demonstrate consistency among the studies, bolstering the credibility of our conclusions. To ensure robust results, we performed meticulous sensitivity checks, publication bias checks, subgroup analysis, and employed the Egger test to detect potential publication bias. Methodological rigor was further ensured by utilizing the Risk of Bias 2.0 tool to evaluate each study. However, our evaluation revealed certain issues with the overall literature. The absence of blinding implementation in the 18 RCTs might be attributed to the inherent challenges of creating a placebo for Xiao Chaihu Decoction, adding complexity to the blinding process. While challenging, blinding is a crucial for ensuring trial fairness and reduce bias. We encourage future research to address these challenges. Additionally, none of the 18 RCTs registered their protocols, leading to a lack of transparency in the trial design, implementation, and completion, which poses a challenge for Podetial replication and potentially contributes to selective reporting bias. (X. X. Li, Han, Wang and Liu, 2013). Moreover, according to GRADE evaluation, most studies were rated as low quality, underscoring of the need for improvement in the quality and methodology of current RCTs. The relatively low sample size analyzed in our study may affect the generalizability and stability of results. Thus, we expect for large-sample studies to further validate our findings. We also recommend that future researchers prioritize implementing blinded and registered clinical trials to enhance the quality and credibility of their studies. In addition, although Xiao Chaihu Decoction is commonly employed in clinical settings and is generally regarded as safe, there are indications from some research studies that it can occasionally lead to interstitial pneumonia, acute hepatitis, and liver damage (Murakami, Okajima, Sakata and Takatsuki, 1995; Hsu, Huang, Tsay, Chang and Lee, 2006; Teschke, 2014). These severe adverse reactions, though rare, underscore the necessity of conducting regular medical examinations to ensure the safe use of Xiao Chaihu Decoction.

## 5 Conclusion

In conclusion, current evidence suggests that Xiao Chaihu Decoction may enhance clinical effectiveness in CRF patients, expedite temperature normalization, ameliorate inflammatory markers, improve quality of life, and minimize adverse reactions. However, to strengthen these findings, future research should focus on conducting high-quality, large-scale RCTs. Additionally, further support for these evidence-based discoveries could be derived from complementary pharmacological experiments.

## Data Availability

The original contributions presented in the study are included in the article/Supplementary Material, further inquiries can be directed to the corresponding author.
