# Supplementary material for: Exploring the therapeutic potential of “Xiaochaihu Decoction”: a systematic review and meta-analysis on the clinical effectiveness and safety in managing cancer-related fever
Source: Front Pharmacol. 2024 May 13;15:1359866. doi: 10.3389/fphar.2024.1359866 (PMC11128760; doi:10.3389/fphar.2024.1359866)
Supplement: Supplementary file 3 [file Table2.docx]

**Appendix B** - Supplementary Table 2 Metabolites identified in Xiaochaihu Decoction by UHPLC-ESI-QTOFMS/MS

N **Identification Formula** Rt(min) Negative Positive

**detected expected Diff**

**MS/MS MS/MS**

source**∆**

|  | | | **Mass** | **Tgt Mass** | **(ppm)** |  | | |
| --- | --- | --- | --- | --- | --- | --- | --- | --- |
| **1 3,4-Dihydroxybenzoic acid** | **C7 H6 O4** | **0.91** | **154.0277** | **154.0266** | **7.11** | **153.0205[M-H]¯,135.0093[M-H-H2O]¯** | **N** | **PT** |
| **2 Caffecic acid*** | **C9 H8 O4** | **2.39** | **180.0426** | **180.0423** | **2.12** | **179.0355[M-H]¯** | **181.0346M+H]+** | **ZJ** |
| **3 Viscidulin I 2'-O-glucoside** | **C21 H20 O12** | **2.74** | **464.0982** | **464.0955** | **5.91** | **463.0910 [M-H]¯, 301.0360 [M-H-glu]¯** | **465.1006[M+H]+,403.1003[[M+H-glu]** | **SB** |
| **4 Zizybeoside I** | **C19 H28 O11** | **2.84** | **432.1657** | **432.1632** | **5.85** | **431.1563[M-H]¯,389.1641[M-H-rha]¯** | **N** | **ZJ** |
| **5 Liquiritigenin 7,4'-diglucoside** | **C27 H32 O14** | **4.33** | **580.1825** | **580.1792** | **5.71** | **625.1785 [M+HCOO]¯, 579.1711[M-H]¯, 417.1194** | **N** | **GU** |
| **6 Liquiritigenin** | **C32 H40 O18** | **5.06** | **712.2248** | **712.2215** | **4.7** | **[M-H-glu]¯ , 255.0672 [M-H-2glu]¯**  **711.2175[M-H]¯, 549.1652[M-H-glu]¯,417.1194** | **713.2155[M+H]+** | **GU** |
| **7-glucoside-4'-apiosyl-(1->2)-glucoside**  **7 Apigenin 6,8 -di-glucoside** | **C27 H30 O15** | **5.31** | **594.1622** | **594.1585** | **6.31** | **[M-H-2glu]¯, 255.0672 [M-H-2glu-api]¯**  **593.1515 [M-H]¯,547.1655 [M-H-CH3]¯, 473.1085** | **595.1516[M+H]+,457.1011[M+H-120-H2O]+** | **SB** |
| **8 Schaftoside** | **C26 H28 O14** | **6.37** | **564.1518** | **564.1479** | **6.95** | **[M-H-120]¯,353.0666 [M-H-240]¯**  **563.1447 [M-H]¯ , 443.0993** | **565.1549[M+H]+,427.0870[M+H-120-H2O]+,2** | **SB** |
|  |  |  |  |  |  | **[M-H-120]¯,353.0671[M-H-240]¯** | **95.0490[M+H-252-H2O]+** |  |
| **9 5,7,2'-Tritydroxy-6-methoxyflavone** | **C16 H12 O6** | **6.38** | **300.0655** | **300.0634** | **7.16** | **345.0617[M+HCOO]¯,** | **301.0738[M+H]+,269.0460,167.0354** | **SB** |
| **10 Quercetin-3-O-glucoside** | **C21 H20 O12** | **6.61** | **464.0980** | **464.0955** | **5.53** | **463.0910 [M-H]¯, 301.0360 [M-H-glu]¯** | **465.1006[M+H]+,403.1003[[M+H-glu]** | **SB** |
| **11 Norwogonin** | **C15 H10 O5** | **6.66** | **270.0543** | **270.0528** | **5.62** | **269.0471 [M-H]¯ , 223.0400 [M-H-H2O-CO]¯** | **271.0589[M+H]+,225.0524[M+H-CO-H2O]+** | **SB** |
| **12 Liquiritin*** | **C21 H22 O9** | **6.76** | **418.1224** | **418.1264** | **9.56** | **417.1221 [M-H]¯,255.0669[M-H-glu]¯** | **419.1225 [M+H]+,257.0679[M+H-glu]+** | **GU** |
| **13 Violanthin** | **C27 H30 O14** | **6.85** | **578.1673** | **578.1636** | **6.5** | **577.1578[M-H]¯, 457.1138 [M-H-120]¯, 337.0727** |  | **SB** |
|  |  |  |  |  |  | **[M-H-240]¯** | **579.1685[M+H]+,441.1137[M+H-120-H2O]+,2** |  |
| **14 Rutin*** | **C27 H30 O16** | **7.31** | **610.1558** | **610.1534** | **3.66** | **609.1471[M-H]¯, 300.0279[M-H-rha-glu]¯** | **57.0747[M+H-252-H2O]+**  **611.1456[M-H]+, 303.0275[M-H-rha-glu]+** | **BC or SB** |
| **15 Chrysin-6-C-beta-D-glucopyranoside-8-C-alpha** | **C26 H28 O13** | **7.41** | **548.1565** | **548.1530** | **6.47** | **547.1483[M-H]¯, 427.1140 [M-H-120]¯, 337.0727** | **549.1593** | **SB** |
| **-L-arabinopyranoside** |  |  |  |  |  | **[M-H-240]¯** | **[M+H]+,411.1010[M+H-120-H2O]+,375.0811[** |  |
|  |  |  |  |  |  |  | **M+H-240-H2O, 279.0600[M+H-252-H2O]+** |  |

| 16 **Chrysin** | **C26 H28 O13** | **7.65** | **548.1568** | **548.1530** | **6.87** | **547.1483[M-H]¯, 427.1140 [M-H-120]¯, 337.0727** | **549.1593** | **SB** |
| --- | --- | --- | --- | --- | --- | --- | --- | --- |
| **6-C-alpha-L-arabinopyranoside-8-C-glucoside** |  |  |  |  |  | **[M-H-240]¯** | **[M+H]+,411.1010[M+H-120-H2O]+,375.0811[** |  |
| 17 **Isomer of Quercetin-3-O-glucoside** | **C21 H20 O12** | **7.76** | **464.0975** | **464.0955** | **4.42** | **463.0910 [M-H]¯, 301.0360 [M-H-glu]¯** | **M+H-240-H2O, 279.0600[M+H-252-H2O]+**  **465.1006[M+H]+,403.1003[[M+H-glu]** | **SB** |
| 18 **Isomer of Violanthin** | **C27 H30 O14** | **7.86** | **578.1660** | **578.1636** | **4.21** | **577.1578[M-H]¯, 457.1138 [M-H-120]¯, 337.0727** |  | **SB** |
|  |  |  |  |  |  | **[M-H-240]¯** | **579.1685[M+H]+,441.1137[M+H-120-H2O]+,2** |  |
| 19 **5,2',6'-Trihydroxy-6,7-dimethoxyflavone** | **C23 H24 O12** | **8.23** | **492.1288** | **492.1268** | **4.01** | **491.1216[M-H]¯, 329.0668[M-H-glu]¯** | **57.0747[M+H-252-H2O]+**  **493.1236[M+H]+, 331.0628[M+H-glu]+** | **SB** |
| **2'-glucoside**  20 **Isomer of** | **C26 H28 O13** | **8.50** | **548.1566** | **548.1530** | **6.49** | **547.1483[M-H]¯, 427.1140 [M-H-120]¯, 337.0727** | **549.1593** | **SB** |
| **Chrysin-6-C-beta-D-glucopyranoside-8-C-alpha** |  |  |  |  |  | **[M-H-240]¯** | **[M+H]+,411.1010[M+H-120-H2O]+,375.0811[** |  |
| **-L-arabinopyranoside**  21 **Carthamidin** | **C15 H12 O6** | **9.02** | **288.0645** | **288.0634** | **3.88** | **287.0572[M-H]¯,269.0410[M-H-H2O]¯,161.0240,125.024** | **M+H-240-H2O, 279.0600[M+H-252-H2O]+**  **N** | **SB** |
| 22 **Chrysin 8-C-glucopyranoside** | **C21 H20 O9** | **9.04** | **416.1119** | **416.1107** | **2.68** | **7**  **415.1054[M-H]¯, 295.0620[M-H-120]¯** | **417.1061[M-H]¯, 297.0624[M-H-120]¯** | **SB** |
| 23 **5,2',6'-Trihydroxy-6,7,8-trimethoxyflavone** | **C24 H26 O13** | **9.42** | **522.1394** | **522.1373** | **3.92** | **521.1322[M-H]¯, 359.0778[M-H-glu]¯** | **523.1329[M+H]¯** | **SB** |
| **2'-glucoside**  24 **Spinosin** | **C28 H32 O15** | **10.61** | **608.1759** | **608.1741** | **2.89** | **607.1687[M-H]¯, 445.0774[M-H-glu]¯, [M-H-glu-rha]¯** | **609.1786[M+H]+,** | **GU** |
| 25 **Liquiritigenin*** | **C15 H12 O4** | **10.65** | **256.0745** | **256.0736** | **3.72** | **255.0658[M-H]¯,135.0080** | **463.1161[M+H-rha]+,301.0659[M+H-rha-glu]+**  **257.0788[M+H]+,242.0580[M-CH3],137.0215** | **GU** |
| 26 **Isoliquiritin*** | **C21 H22 O9** | **11.83** | **418.1280** | **418.1264** | **3.76** | **417.1221 [M-H]¯,255.0669[M-H-glu]¯** | **419.1319[M+H]+,** | **GU** |
| 27 **8-Methoxy-5-O-glucosideflavone** | **C22 H22 O9** | **11.84** | **430.1277** | **430.1264** | **3.02** | **475.1260[M+HCOO]¯,429.2117[M-H]¯, 267.0663[M-H-** | **257.0773[M+H-glu]+,137.0211**  **431.1318[M+H]+,386.2339[M-H-H2O-CO]+,** | **SB** |
| 28 **Licuroside** | **C26 H30 O13** | **12.20** | **550.1700** | **550.1686** | **2.44** | **H2O-CO-glu]¯,**  **549.1628[M-H]¯ , 417.1220[M-H-api]¯,** | **269.0741[M-H- H2O-CO-glu]+,**  **551.1602[M+H]+ , 419.1178[M+H-api]+,** | **GU** |
| 29 **Baicalin*** | **C21 H18 O11** | **12.79** | **446.0867** | **446.0849** | **4.08** | **255.0648[M-H-api-glu]¯**  **445.0789[M-H]¯,** | **257.0712[M+H-api-glu]+,137.018**  **447.1265 [M-H]+,** | **SB** |
|  |  |  |  |  |  | **269.0458[M-H-gluA]¯223.0399[M-H-gluA-H2O-CO]¯** | **271.0527[M-H-gluA]+,123.0399** |  |

| 30 **5,6,7-Trihydroxy-flavanone 7-O-Glucuroside** | **C21 H20 O11** | **13.68** | **448.1020** | **448.1006** | **3.27** | **447.0949[M-H]¯,271.0615[M-H-gluA]¯** | **N** | **SB** |
| --- | --- | --- | --- | --- | --- | --- | --- | --- |
| 31 **Scorzoneroside B** | **C47 H76 O19** | **14.68** | **944.4998** | **944.4981** | **1.86** | **943.4893[M-H]¯,791.4225[M-H-150]¯,629.3595[[M-H-15** | **N** | **BC** |
| 32 **Hydroxyl Oroxylin A-7-O-glucuronide or** | **C22 H20 O12** | **15.25** | **478.1044** | **478.1044** | **1.26** | **0-glu]¯**  **477.1044[M-H]¯,301.0722[M-H-gluA]¯** | **N** | **SB** |
| **hydroxyl wogonoside**  33 **isomer of Baicalin** | **C21 H18 O11** | **15.76** | **446.0866** | **446.0849** | **3.83** | **The same as 29** | **The same as 29** | **SB** |
| 34 **Wogonin 7-glucoside** | **C22 H22 O10** | **16.68** | **446.1221** | **446.1213** | **1.88** | **445.1153[M-H]¯,283.1154[M-H-Glu]¯** | **N** | **SB** |
| 35 **Isomer of Baicalin** | **C21 H18 O11** | **17.21** | **446.0867** | **446.0849** | **4.08** | **The same as 29** | **The same as 29** | **SB** |
| 36 **Scutellarein 4'-methyl ether 7-glucoside** | **C21 H20 O9** | **18.40** | **416.1116** | **416.1107** | **2.09** | **461.1099[M-H]¯,299.0561[M-H-glu]¯.284.0324[M-H-glu-** | **301.0690[M+H]+,286.0456[M+H-CH3]+** | **SB** |
| 37 **Oroxylin A-7-O-glucuronide** | **C22 H20 O11** | **19.24** | **460.1026** | **460.1006** | **4.45** | **CH3]¯**  **459.0975** | **417.1108[M+H]+, 285.0705[M+H-glu]+** | **SB** |
| 38 **Isomer of Baicalin** | **C21 H18 O11** | **20.36** | **446.0867** | **446.0849** | **4.07** | **[M-H]¯,283.0624[M-H-glu]¯.268.0420[M-H-glu-CH3]¯**  **The same as 29** | **The same as 29** | **SB** |
| 39 **Norwogonin** | **C15 H10 O5** | **20.54** | **270.0528** | **270.0536** | **3.02** | **269.0471 [M-H]¯ , 223.0400 [M-H-H2O-CO]¯** | **271.0589[M+H]+,225.0524[M+H-CO-H2O]+** | **SB** |
| 40 **Wogonoside*** | **C22 H20 O11** | **21.31** | **460.1027** | **460.1006** | **4.7** | **459.0975** | **461.1068[M+H]+, 285.0705[M-H-glu]+,** | **SB** |
| 41 **Ginsenoside Rg1*** | **C42 H72 O14** | **22.73** | **800.4954** | **800.4922** | **3.97** | **[M-H]¯,283.0624[M-H-gluA]¯.268.0420[M-H-glu-CH3]¯**  **845.4911[M+HCOO]¯, 799.4886[M-H]¯,** | **270.0473[M-H-glu-CH3]+**  **801.5000[M+H]+ 783.4846[M+H-H2O]+,** | **PG** |
|  |  |  |  |  |  | **637.4275[M-H-Glu]¯, 475.3786[M-H-2Glu]¯** | **603.420590[M+H-glu-2H2O]+,** |  |
| 42 **Jujubasaponin VI** | **C42 H68 O14** | **23.03** | **796.4615** | **796.4609** | **0.77** | **841.4598[M+HCOO]¯,795.4546[M-H]¯,633.4000[M-glu]¯** | **423.3569[]+M+H-2glu-3H2O]+**  **N** | **ZJ** |
| 43 **Ginsenoside Re*or ginsenoside Rd*** | **C48 H82 O18** | **23.26** | **946.5537** | **946.5501** | **3.82** | **991.5508[M+HCOO]¯,** | **970.5394[M+Na+H]+，** | **PG** |
|  |  |  |  |  |  | **945.5423[M-H]¯,799.4866[M-rha]¯, 637.4294** | **795.4546[M-H-glu]+,633.4000[M-2glu]+** |  |
| 44 **4'-hydroxylwogonin** | **C15 H10 O4** | **23.60** | **300.0470** | **300.0437** | **3.3** | **[M-H-rha-glu]¯**  **299.0570[M-H]¯,284.0342[M-H-CH3]¯** | **N** | **SB** |
| 45 **Baicalein*** | **C15 H10 O5** | **24.00** | **270.0539** | **270.0528** | **3.84** | **269.0454[M-H]¯,241.0494[M-H-CO]¯,223.0386[M-H-CO** | **271.0589[M+H]+,225.0524[M+H-CO-H2O]+** | **SB** |
| 46 **Isomer of Liquiritigenin** | **C15 H12 O4** | **25.37** | **256.0741** | **256.0736** | **2.13** | **-H2O],169.0649,136.9878**  **255.0658[M-H]¯,135.0080** | **N** | **GU** |

| 47 **Hydroxysaikosaponin a** | **C42 H70 O14** | **26.59** | **798.4799** | **798.4766** | **4.15** | **843.4785[M-H]¯,** | **N** | **BC** |
| --- | --- | --- | --- | --- | --- | --- | --- | --- |
| 48 **Uralsaponin A** | **C42 H62 O16** | **28.91** | **822.4079** | **822.4038** | **5** | **797.4701[M+HCOO]¯,635.4179[M-H-glu]¯**  **821.400[M-H]¯, 759.4057[M-H-glu]¯** | **823.4021[M+H]+, 613.3674,455.3451,437.3379** | **BC** |
| 49 **1-(4-Hydroxy-3,5-dimethoxyphenyl)-7-(4-hydro** | **C22 H26 O6** | **29.58** | **388.1754** | **388.1729** | **6.39** | **387.1148[M-H]¯, 341.1095[M-H-CO-H2O]¯, 179.0552** | **N** | **ZO** |
| **xy-3-methoxyphenyl)-heptanone**  50 **Ginsenoside Rf*** | **C42 H72 O14** | **30.20** | **800.4968** | **800.4922** | **5.71** | **845.4911[M+HCOO]¯,** | **N** | **PG** |
| 51 **Isomer of ginsenoside Rg1** | **C42 H72 O14** | **30.61** | **800.4955** | **800.4922** | **4.07** | **799.4838[M-H]¯,637.4430[M-glu]¯, 475.3786[M-H-2glu]¯**  **The same as 41** | **N** | **PG** |
| 52 **Wogonin*** | **C16 H12 O5** | **30.73** | **284.0703** | **284.0685** | **6.49** | **283.0631[M-H]¯, 268.0382[M-H-CH3]¯** | **N** | **SB** |
| 53 **Notoginsenosede R2** | **C41 H70 O13** | **31.18** | **770.4852** | **770.4816** | **4.6** | **815.4837 [M+HCOO]¯, 769.4744[M-H]¯, 637.4360** | **N** | **PG** |
| 54 **Chrysin** | **C15 H10 O4** | **31.24** | **254.0591** | **254.0579** | **4.83** | **[M-H-glu]¯**  **253.0518[M-H]¯** | **255.0667[M+H]+,153.0196** | **SB** |
| 55 **Licoricesaponin A3** | **C48 H72 O21** | **31.44** | **984.4626** | **984.4566** | **6.07** | **983.4555[M-H]¯,821.3974[M-H-glu]¯,351.0565[2gluA-]¯** | **985.4678[M+H]+, 810.4329[M-gluA]+,453.3346** | **GU** |
| 56 **Kumatakenin A** | **C17 H14 O6** | **31.58** | **314.0794** | **314.0790** | **1** | **N** | **315.0867[M+H]+, 285.0390** | **GU** |
| 57 **Licoricesaponin G2** | **C42 H62 O17** | **31.90** | **838.4036** | **838.3987** | **5.89** | **837.3932[M-H]¯,661.3716[M-gluA]¯,351.0565[2gluA-]¯** | **839.4073[M+H]+,** | **GU** |
| 58 **Ginsenoside F1** | **C36 H62 O9** | **31.98** | **638.4426** | **638.4394** | **5.04** | **683.4410[M+HCOO]¯, 637.4318[M-H]¯,** | **664[M-gluA]+,469.3303[M-2gluA]+,317.0473**  **N** | **PG** |
| 59 **Ginsenoside Rg2** | **C42 H72 O13** | **32.16** | **784.5017** | **784.4973** | **5.63** | **554.2959,475.3794[M-H-glu]¯**  **829.5008[M+HCOO]¯,783.4931[M-H]¯, 621.4381[M-H-** | **N** | **PG** |
| 60 **Genkwanin** | **C16 H12 O5** | **32.22** | **284.0699** | **284.0685** | **5.11** | **-Glu]¯, 459.3865[M-H- 2Glu]¯**  **283.067¯** | **285.0769[M+H]+,270.0403[M-CH3]+** | **GU** |
| 61 **Saikosaponin s** | **C48 H78 O18** | **32.40** | **942.5237** | **942.5188** | **5.18** | **987.5172[M-HCOO]¯,941.4119[M-H]¯** | **N** | **BC** |
| 62 **Isomer of Ginsenoside F1** | **C36 H62 O9** | **32.41** | **638.4427** | **638.4394** | **5.15** | **683.4410[M+HCOO]¯, 637.4318[M-H]¯,** | **N** | **PG** |
| 63 **Isomer of ginsenoside Rg2** | **C42 H72 O13** | **32.46** | **784.5012** | **784.4973** | **4.97** | **554.2959,475.3794[M-H-glu]¯**  **829.5008[M+HCOO]¯,783.4931[M-H]¯, 621.4381[M-H-** | **N** | **PG** |
| 64 **[6]-Shogaol** | **C17 H24 O3** | **32.60** | **276.1725** | **276.1725** | **-0.13** | **-Glu]¯, 459.3865[M-H- 2Glu]¯**  **N** | **277.1798[M+H]+, 177.0906,145.0645,115.0539** | **ZO** |
| 65 **[6]-Gingerol *** | **C17 H26 O4** | **32.70** | **294.1831** | **294.1831** | **0.11** | **293.1772[M-H]¯,249.1857,193.1608** | **295.1796[M+H],277.1798[M-H2O]+,177.0906,1** | **ZO** |

|  | | | | | | | **45.0645,115.0539** |  |
| --- | --- | --- | --- | --- | --- | --- | --- | --- |
| 66 **Licoricesaponin E2** | **C42 H60 O16** | **32.74** | **820.3938** | **820.3881** | **6.91** | **819.3867[M-H]¯,643.3522[M-H-gluA]¯,** | **N** | **GU** |
|  |  |  |  |  |  | **621.4381[M-H-Glu]¯,** |  |  |
| 67 **Saponin BK1** | **C48 H78 O17** | **32.84** | **926.5296** | **926.5239** | **6.16** | **467.3175[M-H-2Glu]¯,351.0565[2gluA-]¯**  **971.5281[M-H]¯, 925.5175[M-H-CO-H2O]¯,** | **N** | **BC** |
| 68 **5,7-Dihydroxyflavone** | **C19 H18 O8** | **32.87** | **374.1024** | **374.1002** | **5.97** | **779.4587[M-H-Glu]¯**  **373.0952[M-H]¯, 358.0691[M-CH3]¯** | **N** | **SB** |
| 69 **3,5-Diacetoxy-1-(4-hydroxy-3-methoxyphenyl)-7** | **C24 H30 O7** | **32.95** | **430.2011** | **430.1992** | **4.59** | **475.1260[M+HCOO]¯,429.2117[M-H]¯,415.1777[M-CH3** | **N** | **ZO** |
| **-(4-hydroxyphenyl)heptane** |  |  |  |  |  | **]¯,355.1552[M-CH3-CH3COO]¯** |  |  |
| 70 **Isomer of Licoricesaponin G2** | **C42 H62 O17** | **33.09** | **838.4041** | **838.3987** | **6.46** | **The same as 57** | **The same as 57** | **GU** |
| 71 **Ginsenoside Rb1*** | **C54 H92 O23** | **33.17** | **1108.6071** | **1108.6029** | **3.75** | **1107.5985[M-H]¯, 945.5447[M-H-Glu]¯, 622.4453** | **N** | **PG** |
| 72 **Rotundioside D** | **C48 H80 O17** | **33.23** | **928.5459** | **928.5396** | **6.86** | **[M-H-rha-2glu]¯**  **973.5389[M+HCOO]¯, 927.5336[M-H]¯,** | **929.5386[M+H]+** | **BC** |
| 73 **Yunganoside K2** | **C42 H62 O17** | **33.49** | **838.4025** | **838.3987** | **4.47** | **781.4759[M-H--rha]¯**  **The same as 57** | **The same as 57** | **GU** |
| 74 **Floralginsenoside N** | **C53 H90 O22** | **33.52** | **1078.5974** | **1078.5924** | **4.65** | **1123.5961[M+HCOO]¯ 916.5617[M-rha-Glu]¯,** | **N** | **PG** |
| 75 **Isomer of Saponin BK1** | **C48 H78 O17** | **33.61** | **926.5300** | **926.5239** | **6.59** | **783.4878[M-rha-Glu-ara]¯**  **971.5281[M-H]¯, 925.5175[M-H-CO-H2O]¯,** | **N** | **BC** |
| 76 **5-Hydroxy-7,8-dimethoxyflavone** | **C17 H14 O5** | **33.95** | **298.0859** | **298.0841** | **5.93** | **779.4587[M-H- -Glu]¯**  **297.08421[M-H]¯** | **299.0567[M+H]+,153.0190,65.0391** | **SB** |

| 77 **Isomer of Yunganoside K2** | **C42 H62 O17** | **34.03** | **838.4044** | **838.3987** | **6.73** | **The same as 57** | **The same as 57** | **GU** |
| --- | --- | --- | --- | --- | --- | --- | --- | --- |
| 78 **GUcyrrhizic acid*** | **C42 H62 O16** | **34.22** | **822.4094** | **822.4038** | **6.82** | **821.4040[M-H]¯, 645.3699[M-H-gluA]¯,** | **823.4816[M+H]+, 643.4128[M+H-glu-H2O]+** | **GU** |
| 79 **Isomer of Jujubasaponin VI** | **C42 H68 O14** | **34.41** | **796.4652** | **796.4609** | **5.4** | **351.0565[2gluA-]¯**  **841.4598[M+HCOO]¯, 795.4546[M-H]¯,** | **N** | **ZJ** |
| 80 **Isomer of Ginsenoside Re or ginsenoside Rd** | **C48 H82 O18** | **34.64** | **946.5562** | **946.5501** | **6.47** | **633.4000[M-glu]¯**  **991.5508[M+HCOO]¯,** | **N** | **PG** |
|  |  |  |  |  |  | **945.5423[M-H]¯,799.4866[M-rha]¯, 637.4294** |  |  |

|  | | | | | | **[M-H-rha-glu]¯** |  | |
| --- | --- | --- | --- | --- | --- | --- | --- | --- |
| 81 **Isomer of Ginsenoside Rg1** | **C42 H72 O14** | **34.69** | **800.4963** | **800.4922** | **5.12** | **The same as 41** | **The same as 41** | **PG** |
| 82 **Licoricesaponin B2** | **C42 H64 O15** | **34.97** | **808.4298** | **808.4245** | **6.56** | **807.4142[M-H]¯, 631.3900[M-H-gluA]¯,** | **809.4347[M+H]+,** | **GU** |
|  |  |  |  |  |  | **351.0565[2gluA-]¯** | **633.3984[M-gluA]+,439.3550[M-2gluA]+** |  |
| 83 **saikosaponin s** | **C48 H78 O18** | **35.00** | **942.5234** | **942.5188** | **4.82** | **987.5172[M-HCOO]¯, 941.4119[M-H]¯** | **N** | **BC** |
| 84 **Isomer of ginsenoside Rg1** | **C42 H72 O14** | **35.04** | **800.4963** | **800.4922** | **5.12** | **The same as 41** | **N** | **PG** |
| 85 **Isomer of Uralsaponin A** | **C42 H62 O16** | **35.16** | **822.4088** | **822.4038** | **6.12** | **821.400[M-H]¯, 759.4057[M-H-glu]¯** | **N** | **BC** |
| 86 **Saikosaponin a*** | **C42 H68 O13** | **35.27** | **780.4715** | **780.4660** | **7.02** | **825.4699 [M+HCOO]¯, 779.4618[M-H]¯,** | **803.4512[M+Na]+ ,625.1070,441.3697** | **BC** |
| 87 **Sandrosaponin X** | **C48 H76 O19** | **35.38** | **956.5026** | **956.4981** | **4.73** | **617.4062[M-H-glu]¯**  **955.4909 [ M-H]¯** | **957.5082[M+H]+,812.4460[M-rha]+,618.3955** | **BC** |
| 88 **Isomer of Uralsaponin A** | **C42 H62 O16** | **35.55** | **822.4096** | **822.4038** | **7.1** | **821.400[M-H]¯, 759.4057[M-H-glu]¯** | **823.4126[M+H]+, 647.3750[M-gluA]+,453.3346** | **GU** |
| 89 **Saikosaponin b2*** | **C42 H68 O13** | **35.77** | **780.4715** | **780.4660** | **7.02** | **825.4699 [M+HCOO]¯, 779.4618[M-H]¯,** | **N** | **BC** |
| 90 **2'-O-acetyl saikosaponin a** | **C44 H70 O14** | **35.88** | **822.4822** | **822.4766** | **6.87** | **617.4062[M-H-glu]¯**  **867.4811[M+HCOO-],821.4718[M-H],617.4056[M-H-C2** | **845.4689[M+Na]+ ,823.4468[M+H]+,613.3607,** | **BC** |
|  |  |  |  |  |  | **H2O-Glu]** | **437.3433** |  |
| 91 **Isomer of Saikosaponin b2*** | **C42 H68 O13** | **36.04** | **780.4714** | **780.4660** | **6.98** | **825.4699 [M+HCOO]¯, 779.4618[M-H]¯,** | **781.4678[M+H]+,455.3442[M+H-2glu] +** | **BC** |
| 92 **3'-O-acetyl saikosaponin a or4'-O-acetyl** | **C44 H70 O14** | **36.09** | **822.4822** | **822.4766** | **6.87** | **617.4062[M-H-glu]¯**  **The same as 90** | **The same as 90** | **BC** |
| **saikosaponin a**  93 **Prosaikogenin G** | **C36 H58 O8** | **36.24** | **618.4165** | **618.4132** | **5.43** | **663.4148 [ M-H]¯, 617.4037 [ M-H-CO-H2O]¯** | **641.4165[M+Na]+** | **BC** |
| 94 **Saikosaponin d*** | **C42 H68 O13** | **36.32** | **780.4716** | **780.4660** | **7.23** | **825.4699 [M+HCOO]¯, 779.4618[M-H]¯,** | **N** | **BC** |
| 95 **Licoricesaponin C2** | **C42 H62 O15** | **36.40** | **806.4123** | **806.4089** | **4.27** | **617.4062[M-H-glu]¯**  **805.4072[M-H]¯,629.3749[M-H-gluA]¯,** | **N** | **GU** |
| 96 **Isomer of Licoricesaponin B2** | **C42 H64 O15** | **36.61** | **808.4284** | **808.4245** | **4.77** | **453.3424[M-H-2gluA]¯, 351.0565[2gluA-]¯**  **807.4142[M-H]¯,631.3900[M-H-gluA]¯,351.0565[2gluA-]** | **N** | **GU** |
| 97 **6'-O-acetyl saikosaponin a** | **C44 H70 O14** | **36.65** | **822.4808** | **822.4766** | **5.12** | **¯**  **The same as 90** | **The same as 90** | **BC** |

| 98 **Saikosaponin m** | **C42 H68 O12** | **36.70** | **764.4771** | **764.4711** | **7.87** | **809.4699[M+HCOO]¯,763.4650[M-H]¯,601.4140[M-H** | **N** | **BC** |
| --- | --- | --- | --- | --- | --- | --- | --- | --- |
| 99 **Isomer of Prosaikogenin G** | **C36 H58 O8** | **37.23** | **618.4165** | **618.4132** | **5.43** | **-Glu]¯,**  **663.4148 [ M-H]^¯^, 617.4037 [ M-H-CO-H2O]¯** | **N** | **BC** |
| 100 **6'-O-acetyl saikosaponin b2** | **C44 H70 O14** | **37.33** | **822.4827** | **822.4766** | **7.44** | **867.4811[M+HCOO-]¯, 821.4718[M-H] ¯,** | **845.4689,455.3507,437.3398,205.0690** | **BC** |
| 101 **Licoisoflavone B** | **C20 H16 O6** | **37.77** | **352.0949** | **352.0947** | **0.72** | **617.4056[M-H-C2H2O-Glu]¯**  **351.0877[M-H]^-^,323.0586[M-CO]¯** | **N** | **GU** |
| 102 **[8]-Shogaol** |  | **37.77** | **304.2029** | **304.2038** |  | **N** | **305.2056[M+H]， 177.0882,145.0629,117.0686** | **ZO** |
| 103 **2'-O-acetyl saikosaponin ssd** | **C44 H70 O14** | **37.96** | **822.4821** | **822.4766** | **6.74** | **The same as 90** | **N** | **BC** |
| 104 **3'-O-acetyl saikosaponin ssd or 4'-O-acetyl** | **C44 H70 O14** | **38.18** | **822.4831** | **822.4766** | **7.92** | **The same as 90** | **N** | **BC** |
| **saikosaponin ssd or 6'-O-acetyl saikosaponin ssd** |  |  |  |  |  |  |  |  |
| 105 **Isomer of Ginsenoside Rg2** | **C42 H72 O13** | **38.32** | **784.5029** | **784.4973** | **7.13** | **829.5008[M+HCOO]¯,783.4931[M-H]¯, 621.4381[M-H-** | **N** | **PG** |
|  |  |  |  |  |  | **-Glu]¯, 459.3865[M-H- 2Glu]¯** |  |  |
| 106 **Diacetyl saikosponin d** | **C46 H72 O15** | **38.44** | **864.4926** | **864.4871** | **6.37** | **909.4912[M+HCOO]¯,863.4811[M-H]¯,** | **887.4801[M+Na]+,641.4022[M-2C2H2O-2glu]** |  |
|  |  |  |  |  |  | **617.4071[M-H-2CH2CO -glu]¯** | **+,415.1177** |  |
| 107 **Quercetin*** | **C15 H10 O7** | **38.74** | **302.0449** | **302.0427** | **7.39** | **301.0377[M-H]¯, 286.0448[M-H-CH3]¯** | **302.30474912[M+H]+, 258.2740** | **BC or SB** |
| 108 **3'-O-acetyl saikosaponin e** | **C44 H70 O13** | **38.81** | **806.4841** | **806.4816** | **3** | **851.4825[M+HCOO]¯, 805.4744[M-H]¯,** | **807.4188[M+H]+,631.3862,437.3436** | **BC** |
|  |  |  |  |  |  | **745.4555(M-H-CH2CO-H2O), 697.4336,** |  |  |
|  |  |  |  |  |  | **601.4121[M-H-CH2CO-glu]¯** |  |  |
| 109 **(20E)-Ginsenoside F4** | **C42 H70 O12** | **41.05** | **766.4919** | **766.4867** | **6.68** | **811.4866[M+HCOO]¯, 765.4818[M-H]¯, 603.4274** | **N** | **PG** |
|  |  |  |  |  |  | **[M-H-Glu]¯** |  |  |

*Identifications confirmed with pure standards

∆Bupleurum chinense DC. [Apiaceae; Bupleuri radix] (BC), Scutellaria baicalensis Georgi [Lamiaceae; Scutellariae radix] (SB), Pinellia ternata (Thunb.) Makino [Araceae; Pinelliae rhizoma] (PT), Panax ginseng C.A.Mey. [Araliaceae; Ginseng radix et rhizoma] (PG), Ziziphus jujuba Mill. [Rhamnaceae; Jujubae fructus] (ZJ), GUcyrrhiza uralensis Fisch. ex DC. [Fabaceae; GUcyrrhizae radix et rhizoma] (GU), and Zingiber officinale Roscoe [Zingiberaceae; Zingiberis rhizoma recens] (ZO)

N means not detected

Glu =glucosides, gluA= glucuronides, rha= rhamnosyl, ara=arabinosyl, api=apiosyl

In the 2018 study titled "Chemical and Absorption Signatures of Xiao Chai Hu Tang (Xiaochaihu Decoction)," researchers Ting Du and Min Zeng successfully identified 109 metabolites from extracts of Xiaochaihu Decoction. They utilized Ultra-High Performance Liquid Chromatography coupled with Electrospray Ionization Quadrupole Time-of-Flight Mass Spectrometry (UHPLC-ESI Q-TOF MS/MS) to conduct positive and negative ion modes analyses. Identifying these constituents was meticulously done by comparing their UHPLC retention times, accurate masses, and MS/MS fragmentation patterns against available reference standards. This rigorous analytical approach provided a comprehensive chemical profile of the Xiaochaihu Decoction, enhancing our understanding of its potential therapeutic effects [1].

Reference:

[1] Du T, Zeng M, Chen L, Cao Z, Cai H, Yang G. Chemical and Absorption Signatures of Xiao Chai Hu Tang. Rapid communications in mass spectrometry : RCM. 2018. doi.10.1002/rcm.8114.
