# Supplementary material for: Exploring the therapeutic potential of “Xiaochaihu Decoction”: a systematic review and meta-analysis on the clinical effectiveness and safety in managing cancer-related fever
Source: Front Pharmacol. 2024 May 13;15:1359866. doi: 10.3389/fphar.2024.1359866 (PMC11128760; doi:10.3389/fphar.2024.1359866)
Supplement: Supplementary file 4 [file Table5.docx]

| **Appendix F-** ConPhyMP checklist of items for conducting and reporting analytical  methods1,2 relevant for extract type A (for species or botanical drugs covered  in a monograph in one of the national or regional pharmacopoeias) | | | | | |
| --- | --- | --- | --- | --- | --- |
| Study ID | **SSECTION/TOPIC**  *Type of extract* | **SECTION/TOPIC**  *Preferred/main methods for*  *extract characterisation/*  *chemical analysis* | **SECTION/TOPIC**  *Alternative methods for extract*  *characterisation/chemical analysis* | **SECTION/TOPIC**  *Use of reference standards* | **SECTION/TOPIC**  *Comparison of different extracts/*  *samples of the same plants* |
|  | **CHECKLIST ITEM-1** | **CHECKLIST ITEM-2** | **CHECKLIST ITEM-3** | **CHECKLIST ITEM-4** | **CHECKLIST ITEM-5** |
| Xiao X 2022 | Yes(Page 1) | No | No | No | No |
| Gong SX 2020 | Yes(Page 1) | No | No | No | No |
| Ma L 2020 | Yes(Page 1) | No | No | No | No |
| Hu JN 2020 | Yes(Page 1) | No | No | No | No |
| Lin MB 2020 | Yes(Page 1) | No | No | No | No |
| Chen YH 2019 | Yes(Page 1) | No | No | No | No |
| Zhang J 2017 | Yes(Page 1) | No | No | No | No |
| Zhu ZC 2017 | Yes(Page 1) | No | No | No | No |
| Song YL 2017 | Yes(Page 1) | No | No | No | No |
| Wu JS 2016 | Yes(Page 1) | No | No | No | No |
| Luo SJ 2015 | Yes(Page 1) | No | No | No | No |
| Li H 2014 | Yes(Page 1) | No | No | No | No |
| Xu XY 2013 | Yes(Page 1) | No | No | No | No |
| Dai CS 2013 | Yes(Page 1) | No | No | No | No |
| Li S 2013 | Yes(Page 1) | No | No | No | No |
| Zheng QH 2010 | Yes(Page 1) | No | No | No | No |
| Ma CZ 2002 | Yes(Page 1) | No | No | No | No |
| Peng SW 2018 | Yes(Page 1) | No | No | No | No |

**CHECKLIST ITEM-1，-Confirm that the species or botanical drug under investigation iscovered in a monograph in one of the national or regionalpharmacopoeias. CHECKLIST ITEM-2，Aompliance with pharmacopoeial standards to be followed:(a)The description ofthe active ingredients in the botanical drug (if known) or analytical marker compounds as defined. (b)An analysis as defined in the monograph is needed if the extract has not been supplied with a certificate. (c) lf the preparation was purchased, the manufacturer and certificateof analysis need to be included. Including either the preferred or alternative approaches forcharacterisation: (a) Triple chemical fingerprinting methods, each with one or more detection parameters. (b)Quantification ofat least two marker compounds (unless this is notfeasible,evidence needs to be provided), and justification of thechoice of markers (if applicable). CHECKLIST ITEM-3，(a)Single chemical fingerprinting method with at least three differentdetection parameters (i.e., altered detection parameters, like TLCHPTLC with different staining reagents and/or UV excitation wavelengths, HPLC-DAD/LCDAD with different wavelengths). The sameapplies to coupling MS or NMR to chromatographic techniques.(b)Quantification of at least two marker compounds (unless this is notfeasible, evidence needs to be provided), and justification of thechoice ofmarkers (if applicable). CHECKLIST ITEM-4，(a) Direct overlay of the chromatogram of the sample with that of anofficially specified reference standard (if applicable). (b)Chromatographic fingerprinting: Direct overlay of the chromatogram of the sample with that of official reference standards of thepowdered plant material or the dry extract from the plant material. CHECKLIST ITEM-5，(a)Direct comparison ofthe chromatographic/spectroscopic systemand/or scoring system for "similarity" to be followed.Yes, reported, No, not reported.**

**Reference:**

**1 Please acknowledge/cite this as follows: Heinrich M, Jalil B, Abdel-Tawab M, Echeverria J, Kulić Ž, McGaw LJ, et al. Best Practice in the chemical characterisation of extracts used in pharmacological and toxicological research—**

**The ConPhyMP—Guidelines. Frontiers in Pharmacology. 2022;13:953205. https://doi.org/10.3389/fphar.2022.953205**

**2 We strongly recommend reading this checklist in conjuncton with ConPhyMP 2022 explanaton and elaboraton for important clarifications on all items. If relevant, we also recommend after reading Heinrich et al. (2020) Best**

**practice in research—Overcoming common challenges in phytopharmacological research. Journal of Ethnopharmacology. 2020;246:112230. https://doi.org/10.1016/j.jep.2019.112230**
