# Supplementary material for: Exploring the therapeutic potential of “Xiaochaihu Decoction”: a systematic review and meta-analysis on the clinical effectiveness and safety in managing cancer-related fever
Source: Front Pharmacol. 2024 May 13;15:1359866. doi: 10.3389/fphar.2024.1359866 (PMC11128760; doi:10.3389/fphar.2024.1359866)
Supplement: Supplementary file 5 [file Table3.docx]

Appendix C

**The search strategy for the respective database.**

| Database name | Search strategies |
| --- | --- |
| CNKI | TKA=('柴胡') AND TKA=('发热'+'潮热'+'低热'+'烦热'+'寒热往来'+'壮热') AND TKA=('癌'+'瘤') AND FT='随机' |
| Wanfang | 主题:("柴胡" ) and 主题:("发热"or "潮热" or "低热" or"烦热"or "寒热往来"or "壮热") and 主题:("癌" or "瘤") and 全部:(随机) |
| VIP | (M=(柴胡) or K=(柴胡)) and (M=(发热 or 潮热 or 低热 or 烦热 or 寒热往来 or 壮热) or K=(发热 or 潮热 or 低热 or 烦热 or 寒热往来 or 壮热)) and (M=(癌 or 瘤) or K=(癌 or 瘤)) and U=随机 |
| SinoMed | 1 “柴胡”[不加权:扩展]  2 “肿瘤”[不加权:扩展]  3 “发热”[不加权:扩展]  4 “柴胡”[常用字段:智能]  5 “发热”[常用字段:智能] OR “潮热”[常用字段:智能] OR “往来寒热”[常用字段:智能] OR “低热”[常用字段:智能] OR “烦热”[常用字段:智能] OR “壮热”[常用字段:智能]  6 “瘤”[常用字段:智能] OR “癌”[常用字段:智能]  7 (#1) OR (#4)  8 (#2) OR (#6)  9 (#3) OR (#5)  10 (#7) AND (#8) AND (#9)  11 “随机”[全部字段:智能]  12 (#10) AND (#11) |
| Pubmed | #1"Bupleurum"[Mesh]  #2((((((Bupleurums[Title/Abstract]) ) OR (Chai Hu[Title/Abstract])) OR (Saiko[Title/Abstract])) OR (Hare's Ear[Title/Abstract])) OR (Ear, Hare's[Title/Abstract])) OR (Hare's Ears[Title/Abstract])  #3(((((((Bupleurums[Title/Abstract]) ) OR (Chai Hu[Title/Abstract])) OR (Saiko[Title/Abstract])) OR (Hare's Ear[Title/Abstract])) OR (Ear, Hare's[Title/Abstract])) OR (Hare's Ears[Title/Abstract])) OR ("Bupleurum"[Mesh])  #4"Fever"[Mesh]  #5(((Fever[Title/Abstract]) OR (Fevers[Title/Abstract])) OR (Pyrexia[Title/Abstract])) OR (Pyrexias[Title/Abstract])  #6((((Fever[Title/Abstract]) OR (Fevers[Title/Abstract])) OR (Pyrexia[Title/Abstract])) OR (Pyrexias[Title/Abstract])) OR ("Fever"[Mesh])  #7(((((Fever[Title/Abstract]) OR (Fevers[Title/Abstract])) OR (Pyrexia[Title/Abstract])) OR (Pyrexias[Title/Abstract])) OR ("Fever"[Mesh])) AND ((((((((Bupleurums[Title/Abstract]) ) OR (Chai Hu[Title/Abstract])) OR (Saiko[Title/Abstract])) OR (Hare's Ear[Title/Abstract])) OR (Ear, Hare's[Title/Abstract])) OR (Hare's Ears[Title/Abstract])) OR ("Bupleurum"[Mesh]))  #8"Neoplasms"[Mesh]  #9(((((((((((((((((cancer[Title/Abstract]) OR (Tumor[Title/Abstract])) OR (Neoplasm[Title/Abstract])) OR (Tumors[Title/Abstract])) OR (Neoplasia[Title/Abstract])) OR (Neoplasias[Title/Abstract])) OR (Cancers[Title/Abstract])) OR (Malignant Neoplasm[Title/Abstract])) ) OR (Malignancy[Title/Abstract])) OR (Malignancies[Title/Abstract])) OR (Malignant Neoplasms[Title/Abstract])) OR (Neoplasm, Malignant[Title/Abstract])) OR (Neoplasms, Malignant[Title/Abstract])) OR (Benign Neoplasms[Title/Abstract])) OR (Benign Neoplasm[Title/Abstract])) OR (Neoplasms, Benign[Title/Abstract])) OR (Neoplasm, Benign[Title/Abstract])  #10((((((((((((((((((cancer[Title/Abstract]) OR (Tumor[Title/Abstract])) OR (Neoplasm[Title/Abstract])) OR (Tumors[Title/Abstract])) OR (Neoplasia[Title/Abstract])) OR (Neoplasias[Title/Abstract])) OR (Cancers[Title/Abstract])) OR (Malignant Neoplasm[Title/Abstract])) ) OR (Malignancy[Title/Abstract])) OR (Malignancies[Title/Abstract])) OR (Malignant Neoplasms[Title/Abstract])) OR (Neoplasm, Malignant[Title/Abstract])) OR (Neoplasms, Malignant[Title/Abstract])) OR (Benign Neoplasms[Title/Abstract])) OR (Benign Neoplasm[Title/Abstract])) OR (Neoplasms, Benign[Title/Abstract])) OR (Neoplasm, Benign[Title/Abstract])) OR ("Neoplasms"[Mesh])  #11(((((((((((((((((((cancer[Title/Abstract]) OR (Tumor[Title/Abstract])) OR (Neoplasm[Title/Abstract])) OR (Tumors[Title/Abstract])) OR (Neoplasia[Title/Abstract])) OR (Neoplasias[Title/Abstract])) OR (Cancers[Title/Abstract])) OR (Malignant Neoplasm[Title/Abstract])) ) OR (Malignancy[Title/Abstract])) OR (Malignancies[Title/Abstract])) OR (Malignant Neoplasms[Title/Abstract])) OR (Neoplasm, Malignant[Title/Abstract])) OR (Neoplasms, Malignant[Title/Abstract])) OR (Benign Neoplasms[Title/Abstract])) OR (Benign Neoplasm[Title/Abstract])) OR (Neoplasms, Benign[Title/Abstract])) OR (Neoplasm, Benign[Title/Abstract])) OR ("Neoplasms"[Mesh])) AND ((((((Fever[Title/Abstract]) OR (Fevers[Title/Abstract])) OR (Pyrexia[Title/Abstract])) OR (Pyrexias[Title/Abstract])) OR ("Fever"[Mesh])) AND ((((((((Bupleurums[Title/Abstract]) ) OR (Chai Hu[Title/Abstract])) OR (Saiko[Title/Abstract])) OR (Hare's Ear[Title/Abstract])) OR (Ear, Hare's[Title/Abstract])) OR (Hare's Ears[Title/Abstract])) OR ("Bupleurum"[Mesh]))) |
| Cochrane Library | #1 TS=(Bupleurums OR Chai Hu OR Saiko OR Hare's Ear OR Ear, Hare's OR Hare's Ears OR Bupleurum)  #2 TS=(Fever OR Fevers OR Pyrexia OR Pyrexias OR "Fever")  #3 TS=(cancer OR Tumor OR Neoplasm OR Tumors OR Neoplasia OR Neoplasias OR Cancers OR Malignant Neoplasm OR Malignancy OR Malignancies OR Malignant Neoplasms OR Neoplasm, Malignant OR Neoplasms, Malignant OR Benign Neoplasms OR Benign Neoplasm OR Neoplasms, Benign OR Neoplasm, Benign OR "Neoplasms")  #4 #1 AND #2 AND #3 |
| Embase | #1 ‘bupleurum’/exp  #2 thorowax:ab,ti OR bupleurum:ab,ti  #3 #1 OR #2  #4 ‘fever’/exp  #5 ‘body temperature elevation’:ab,ti OR ‘febrile disease’:ab,ti OR ‘febrile reaction’:ab,ti OR ‘febrile response’:ab,ti OR pyrexia:ab,ti OR ‘sweating sickness’:ab,ti OR fever:ab,ti  #6 #4 OR #5  #7 ‘neoplasm’/exp  #8 ‘acral tumor’:ab,ti OR ‘acral tumor’:ab,ti OR neoplasia:ab,ti OR neoplasms:ab,ti OR ‘neoplastic disease’:ab,ti OR ‘neoplastic entity’:ab,ti OR ‘neoplastic mass’:ab,ti OR tumor:ab,ti OR ‘tumoral entity’:ab,ti OR ‘tumoral mass’:ab,ti OR ‘tumorous entity’:ab,ti OR ‘tumorous mass’:ab,ti OR tumors:ab,ti OR tumour:ab,ti OR ‘tumoural entity’:ab,ti OR ‘tumoural mass’:ab,ti OR ‘tumourous entity’:ab,ti OR ‘tumourous mass’:ab,ti OR tumours:ab,ti OR neoplasm:ab,ti  #9 #7 OR #8  #10 #3 AND #4 AND # 9 |
| Web of Science | #1 MeSH descriptor [Bupleurum] explode all trees  #2 Bupleurum OR Synonyms OR Saiko OR Bupleurums OR Ear,Hare’s OR Hare’s Ear OR Hare’s Ears OR Chai Hu  #3 MeSH descriptor:[Fever] explode all trees  #4 Fever OR Synonyms OR Pyrexia OR Fevers OR Pyrexias  #5 MeSH descriptor:[Neoplasms] explode all trees  #6 Neoplasms OR Synonyms:Neoplasm OR Neoplasias OR Tumors OR Neoplasia OR Tumor OR Benign Neoplasms OR Neoplasm,Benign OR Neoplasms,Benign OR Benign Neoplasm OR Cancers OR Neoplasms,Malignant OR Malignant Neoplasms OR Neoplasm,Malignant OR Malignancy OR Malignancies OR Malignant  #7 #1 OR #2  #8 #3 OR #4  #9 #5 OR #6  #10 #7 AND #8 AND #9  #11 random  #12 #10 AND #11 |

CNKI: China National Knowledge Infrastructure; SinoMed: the Chinese Biomedical Literature Database; WanFang: the WanFang Database; VIP: the Chinese Scientific Journals Full-Text Database; Embase Database: Excerpta Medica Database; WOS Database: Web of Science Database.
