# Supplementary material for: Exploring the therapeutic potential of “Xiaochaihu Decoction”: a systematic review and meta-analysis on the clinical effectiveness and safety in managing cancer-related fever
Source: Front Pharmacol. 2024 May 13;15:1359866. doi: 10.3389/fphar.2024.1359866 (PMC11128760; doi:10.3389/fphar.2024.1359866)
Supplement: Supplementary file 6 [file Table4.docx]

**Appendix D**- The specific gram weights of each botanical ingredient for a single dose of Xiaochaihu Decoction, as recorded in various studies.

| Study ID | *Bupleurum chinense DC* | *Scutellaria baicalensis Georgi* | *Pinellia ternata (Thunb.) Makino* | *Zingiber officinale Roscoe* | *Panax ginseng C.A.Mey.* | *Ziziphus jujuba Mill.* | *Glycyrrhiza uralensis Fisch. ex DC.* |
| --- | --- | --- | --- | --- | --- | --- | --- |
| Xiao X 2022 | 15g | 15g | 10g | 9g | 24g | 0g | 10g |
| Gong SX 2020 | 12g | 12g | 10g | 3g | 10g | 15g | 3g |
| Ma L 2020 | 15g | 30g | 10g | 10g | 10g | 12g | 10g |
| Hu JN 2020 | 15g | 15g | 10g | 9g | 9g | 15g | 9g |
| Lin MB 2020 | 15g | 9g | 9g | 3g | 9g | 36g | 6g |
| Chen YH 2019 | 12g | 12g | 10g | 3g | 10g | 15g | 3g |
| Zhang J 2017 | 15g | 30g | 10g | 10g | 6g | 15g | 10g |
| Zhu ZC 2017 | 30g | 12g | 12g | 9g | 12g | 18g | 9g |
| Song YL 2017 | 15g | 12g | 10g | 3g | 10g | 15g | 3g |
| Wu JS 2016 | 12g | 12g | 10g | 3g | 10g | 15g | 3g |
| Luo SJ 2015 | 20g | 10g | 20g | 10g | 15g | 15g | 10g |
| Li H 2014 | 20g | 10g | 20g | 10g | 15g | 15g | 10g |
| Xu XY 2013 | 12g | 12g | 10g | 3g | 10g | 15g | 3g |
| Dai CS 2013 | 20g | 15g | 10g | 0g | 10g | 15g | 5g |
| Li S 2013 | 12g | 12g | 10g | 3g | 10g | 15g | 3g |
| Zheng QH 2010 | 12g | 12g | 10g | 3g | 10g | 15g | 3g |
| Ma CZ 2002 | NR | NR | NR | NR | NR | NR | NR |
| Peng SW 2018 | 24g | 9g | 9g | 9g | 9g | 3g | 9g |
| ***Average(not included Ma CZ 2002)*** | 16.24g | 14.06g | 11.18g | 5.88g | 11.12g | 14.65g | 6.41g |
