# Supplementary material for: Exploring the therapeutic potential of “Xiaochaihu Decoction”: a systematic review and meta-analysis on the clinical effectiveness and safety in managing cancer-related fever
Source: Front Pharmacol. 2024 May 13;15:1359866. doi: 10.3389/fphar.2024.1359866 (PMC11128760; doi:10.3389/fphar.2024.1359866)
Supplement: Supplementary file 7 [file DataSheet1.docx]

Appendix E

S1


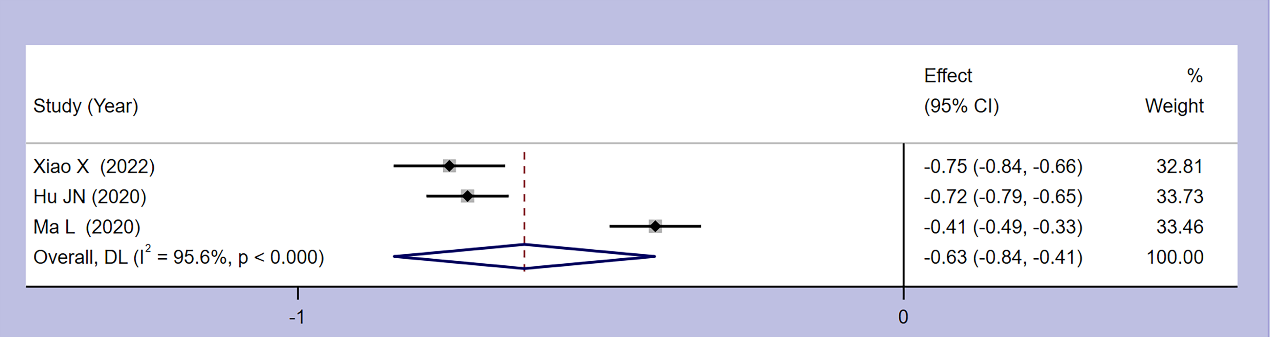
A


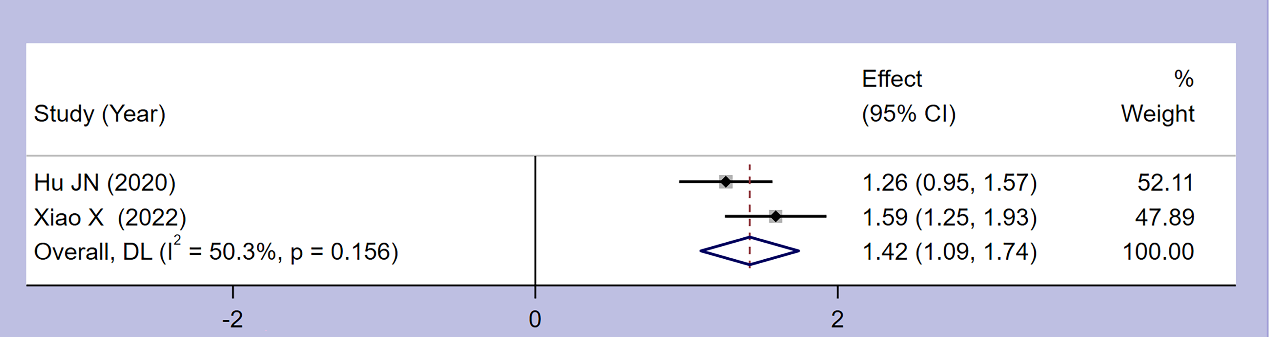
B

Fig. S1. Forest plot of the meta-analysis on the inflammatory cytokines levels. (A) TNF-α levels. (B) IL-2 levels. MD, mean difference. CI: confidence interval.

S2


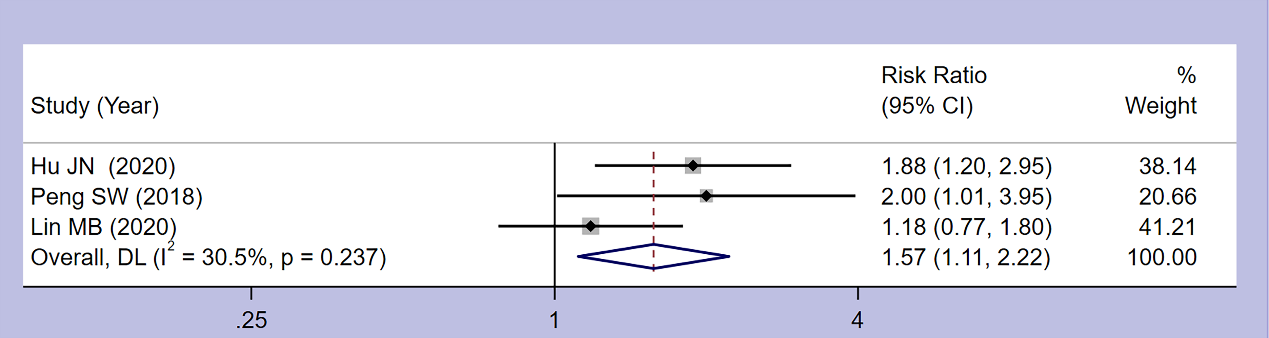


Fig. S2. Forest plot of the meta-analysis on the Karnofsky Performance Status (KPS) score RR: risk ratio, CI: confidence interval.

S3


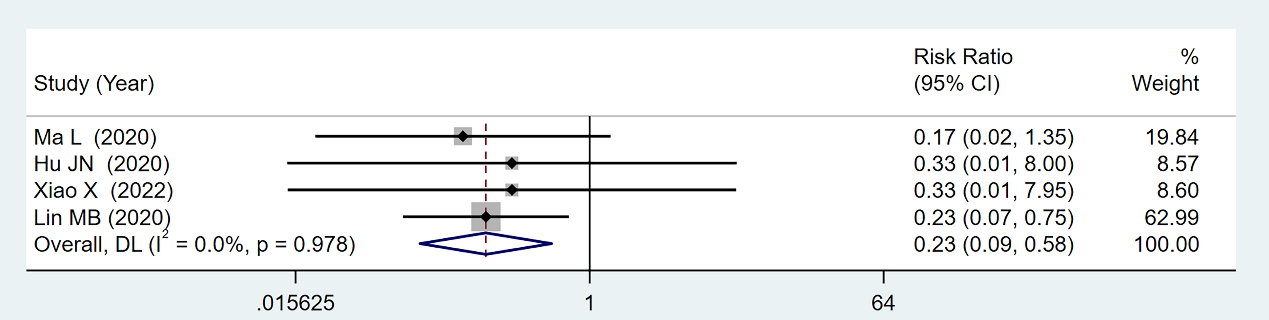
A

B
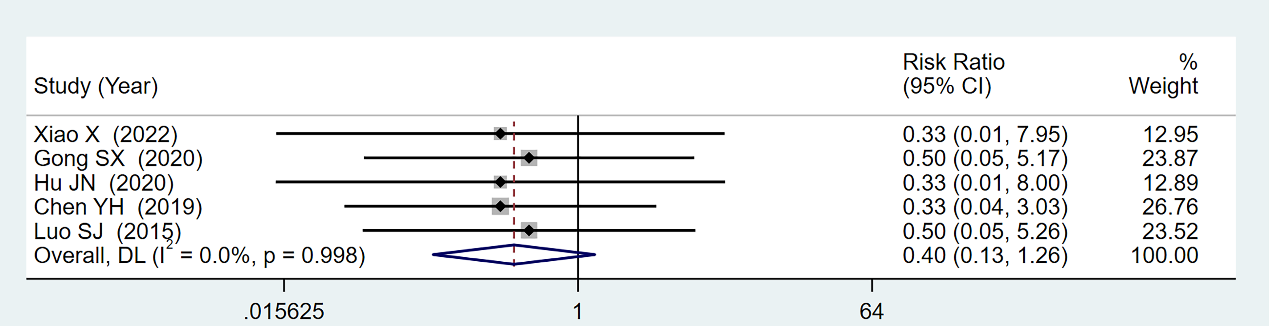


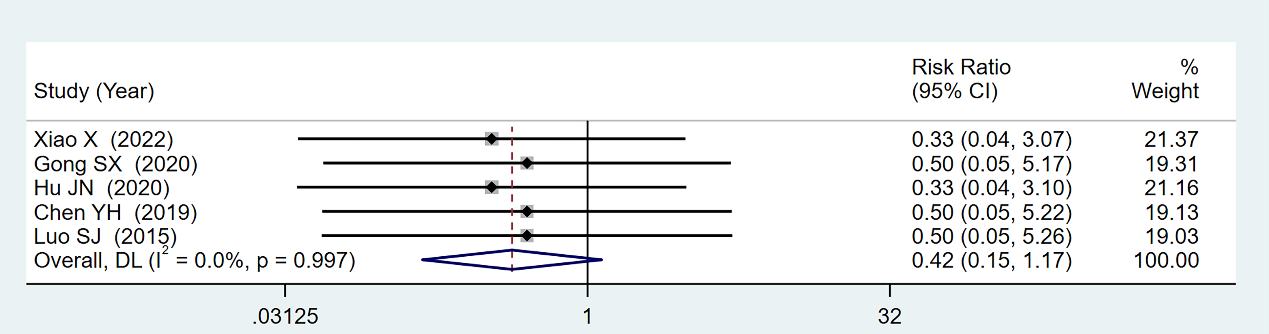
C

Fig. S3. Forest plot of the meta-analysis on the adverse events. RR: risk ratio, CI: confidence interval. (A) The incidence of abdominal bloating/pain. (B) The incidence of palpitations. (C) dizziness/headache.

S4


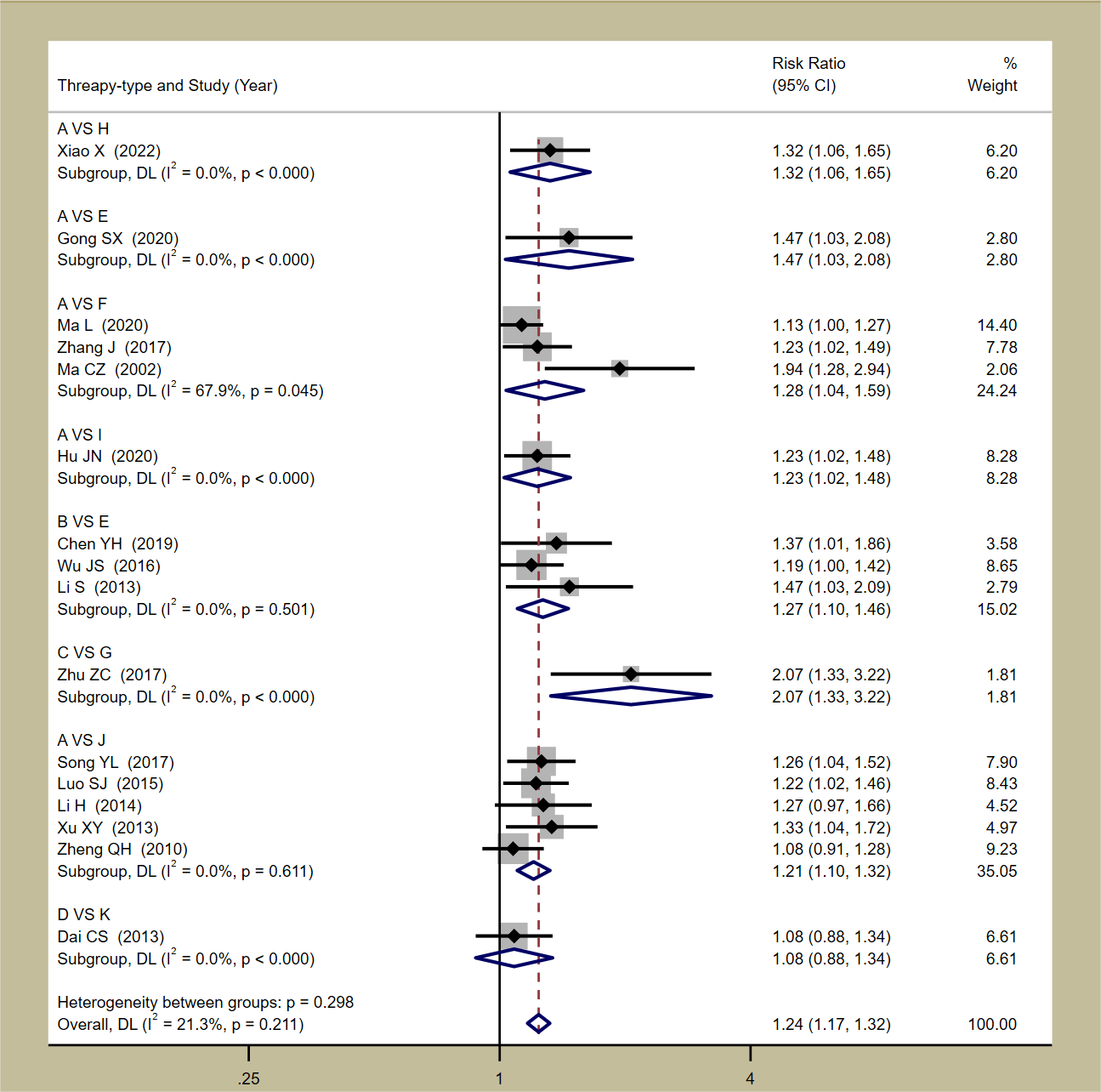


**A**


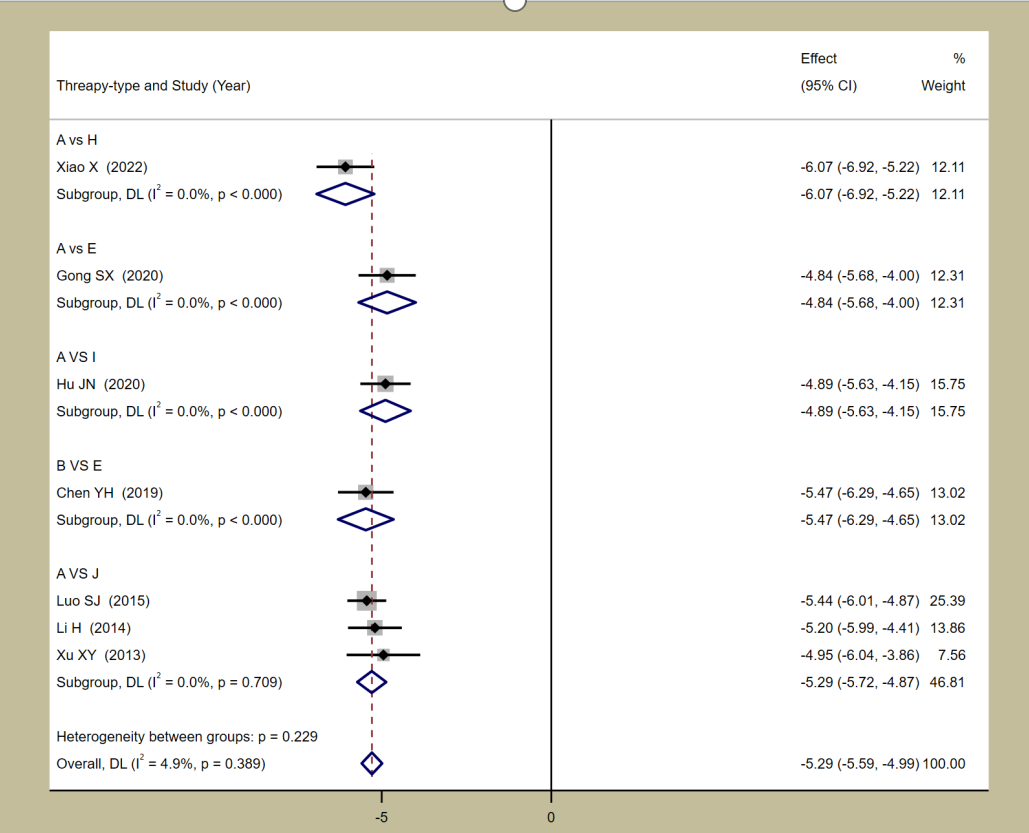


B

Fig. S4. The results of the subgroup analysis. RR: risk ratio, MD: mean difference, CI: confidence interval. (A) Clinical effectiveness. (B) Time to normalize body temperature. The specific meanings of all treatment measures are as follows: A, Xiaochaihu Decoction; B, Xiaochaihu Decoction plus Indomethacin; C, Xiaochaihu Decoction plus Xinhuang Tablets; D, Xiaochaihu Decoction plus radiotherapy and chemotherapy; E, Indomethacin; F, Naproxen Tablets; G, Xinhuang Tablets; H, Ibuprofen plus lysine-aspirin; I, Ibuprofen plus lysine-aspirin plus Indomethacin; J, Antipyretics plus antibiotic; K, Radiotherapy plus chemotherapy plus Indomethacin; L, Pure radiotherapy plus chemotherapy.

S5


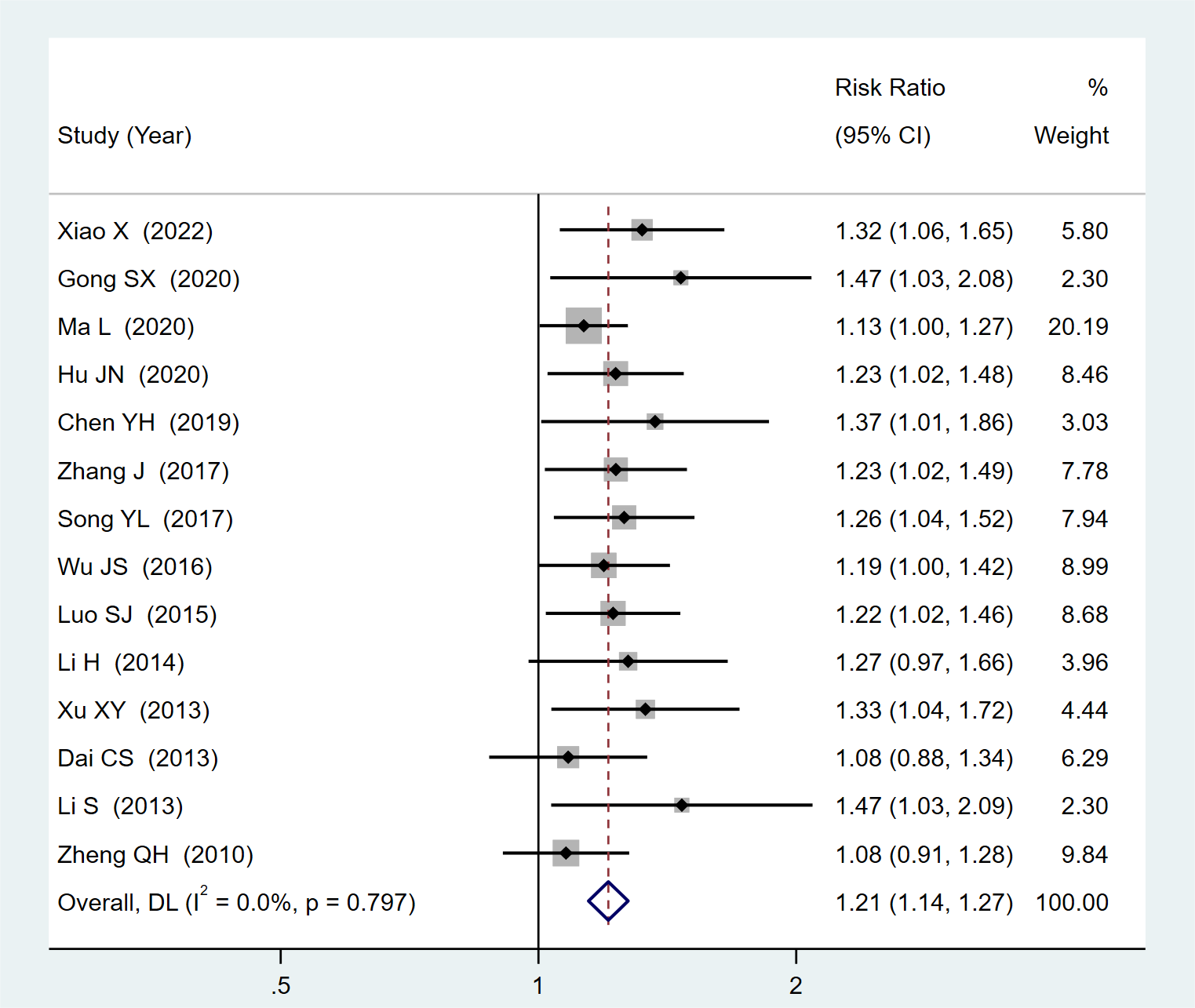


Fig. S5. Forest plot of the clinical effectiveness after removing two studies. RR: risk ratio, CI: confidence interval.
